# Novel Roles for the Ectoenzyme CD38 in the Maintenance of Transcriptional and Metabolic Homeostasis in Astrocytes

**DOI:** 10.1002/glia.70112

**Published:** 2025-12-16

**Authors:** S. Basak, A. M. Colafrancesco, R. D. Hernandez, X. Wei, L. J. McMeekin, D. Dang, M. Simmons, J. L. Browning, K. Noel, F. Zhou, F. E. Lund, J. S. Saad, S. G. Watsen, A. M. Pickrell, R. M. Cowell, M. L. Olsen

**Affiliations:** ^1^ School of Neuroscience, Virginia Tech Blacksburg Virginia USA; ^2^ Translational Biology Medicine and Health Graduate Program, Virginia Tech Blacksburg Virginia USA; ^3^ Department of Neurology University of Alabama at Birmingham Birmingham Alabama USA; ^4^ Department of Human Genetics Emory University Atlanta Georgia USA; ^5^ Department of Neuroscience Southern Research Birmingham Alabama USA; ^6^ Department of Microbiology University of Alabama at Birmingham Birmingham Alabama USA

**Keywords:** aging, astrocyte, brain metabolism, mitochondria, neurodegeneration, RNA‐sequencing

## Abstract

CD38 is an ectoenzyme that converts NAD+ to NAM to help maintain bioenergetic homeostasis. CD38 dysregulation and gene variation is reported in neurodegenerative conditions such as Parkinson's disease (PD) and Alzheimer's disease (AD), highlighting the need to better understand CD38 biology within the brain. Here, we demonstrate enrichment of *Cd38* in midbrain astrocytes and describe how CD38 deficiency influences brain metabolism, astrocytic gene expression, and bioenergetics. We demonstrate increased NAD content, decreased NAM content, and increased NAD/NAM in the midbrain and striatum of CD38‐deficient (*Cd38* ^*−/−*^) mice, indicating the dependence on CD38 for NAD to NAM conversion in the brain. RNA‐sequencing of isolated astrocytes revealed numerous differentially expressed genes in *Cd38* ^*+/−*^ and *Cd38* ^*−/−*^ mice, with alterations in mitochondrial, metabolic, senescence‐related, astrocyte reactivity, and other genes involved in PD and AD etiology. Furthermore, functional metabolic analysis of midbrain revealed changes in pyruvate oxidation, age‐dependent increase of citrate synthase (CS) activity, and reduction of cytochrome c oxidase‐to‐CS ratio in *Cd38* deficiency. These findings identify a novel role for astrocytes in the regulation of CD38‐dependent NAD/NAM homeostasis in the brain and provide a framework for future studies evaluating the relationship between CD38 dysfunction, aging, and vulnerability of neuronal populations in neurodegenerative disease. Importantly, these studies underscore the necessity to better resolve the impact of CD38 deficiency on brain metabolism, considering ongoing clinical trials and discussions related to the use of CD38 modulators for the treatment of cancers, age‐related decline, and neurodegenerative disease.

## Graphical abstract

- CD38 deficiency influences brain NAD/NAM metabolism.
- Global CD38 deficiency drives changes in astrocyte mitochondrial, metabolic, senescence, and neurodegenerative‐related genes.
- CD38 deficiency alters glial reactivity and astrocyte bioenergetics.

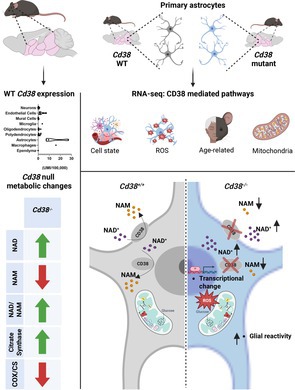

## 1 Introduction

*CD38* encodes the Cluster of Differentiation 38 (CD38) protein, an ectoenzyme that plays a vital role in bioenergetics and inflammatory responses (Chini et al. 2020; Hogan et al. 2019). CD38 utilizes nicotinamide adenine dinucleotide (NAD+) as a substrate to produce nicotinamide mononucleotide (NAM), adenine diphosphate ribose (ADPR), and cyclic‐ADP‐ribose (cADPR), in both extracellular and intracellular compartments. NAD+ is an essential coenzyme primarily regarded for its use in oxidation–reduction reactions to generate mitochondrial ATP. While NAD is not cell permeable, NAM can readily cross the plasma membrane and be utilized to generate NAD+ through salvage pathways (Covarrubias et al. 2021; Lautrup et al. 2019). In typical contexts, NAD generation and re‐generation are balanced in a way that stabilizes intracellular REDOX status, bioenergetics, and other downstream pathways. The aging process often disrupts this balance and favors CD38 overactivation and NAD insufficiency (Covarrubias et al. 2021) which may impair mitochondrial ATP production and cellular bioenergetics (Lautrup et al. 2019).

In the brain, CD38 is largely expressed by glial cells (Kelley et al. 2018), with the highest expression observed in astrocytes (Hattori et al. 2023; Hattori et al. 2017). Astrocytic CD38 has been demonstrated to play a role in glial development (Hattori et al. 2017), neuroinflammatory modulation (Guerreiro et al. 2020) and mitochondrial transfer (Hayakawa et al. 2016). Although CD38 expression is thought to rise with age and deplete NAD+ supplies (Camacho‐Pereira et al. 2016), its role in neurodegeneration remains poorly understood due to conflicting findings. Some results suggest that deletion of CD38 is neuroprotective, as decreased plaque loads and improved spatial learning are reported in Alzheimer's disease (AD) models (Blacher et al. 2015); however, contradictory reports of cognitive deficits (Kim et al. 2016) and increased susceptibility to infection (Lischke et al. 2013), which may be due in part to the ability of CD38 to regulate inflammation and the innate immune response (Partida‐Sanchez et al. 2001), are also found in *Cd38* knockout mice. CD38 is also implicated in other age‐related neurodegenerative diseases like Parkinson's disease (PD), whereby *CD38* polymorphisms result in an increased risk of PD development (Chang et al. 2017). For example, *BST1*/*CD157* polymorphism is a paralog gene of *CD38* that has been implicated as a risk factor for PD (Guerreiro et al. 2020). Due to the high energetic demands of dopaminergic neurons typically lost in PD, it is possible that CD38's regulatory role in NAD+ bioavailability is responsible for maintaining the energetic balance necessary for neuronal survival. Supporting this, iPSC neurons from PD patients show altered NAD+ metabolism with imbalanced NAD+/NAM ratios (Schondorf et al. 2018) similar to those reported for *Cd38* knockout mice.

While many studies have focused on elucidating the biological roles for genes causative for familial PD, monogenic causes of PD only account for approximately 5%–15% (Skrahina et al. 2021; Vollstedt et al. 2023) of all cases, indicating that it is imperative to explore how environmental factors and non‐causal risk genes contribute to PD pathology. Human genome‐wide association studies (GWAS) have identified potential risk genes associated with the development of PD (Nalls et al. 2019; Yao et al. 2021; Zhou et al. 2023). In a recent publication, researchers compiled multiple human transcriptomic and proteomic datasets for a meta‐analysis to identify targets for treatments and further study (Zhou et al. 2023). Extensive correlation analysis identified *GPNMB* and *CD38* expression as the strongest indicators of PD risk. Even further, human‐based Mendelian randomization studies have identified single‐nucleotide polymorphisms (SNPs) in the *CD38* locus that confer increased risk of PD (Chen et al. 2014; Li et al. 2019; Sharma et al. 2012; Storm et al. 2021). However, it is not clear how alterations in the expression or function of CD38 could contribute to brain dysfunction and disease.

Here we sought to evaluate how *CD38* deficiency influences brain metabolism, astrocytic gene expression, and bioenergetics with the goal of characterizing cellular dysfunction most robustly affected by CD38 deficiency. Herein, we describe how CD38 deficiency influences bioenergetics, astrocyte gene expression, and mitochondrial function. We utilized wildtype (*Cd38* ^*+/+*^), heterozygous *Cd38* (*Cd38* ^*+/−*^), and knockout *Cd38* (*Cd38* ^*−/−*^) mice to evaluate transcriptional, metabolic, and functional changes associated with CD38 deficiency in the midbrain, a region enriched in dopaminergic neurons, which are vulnerable to dysfunction and cell loss in PD. We found that astrocytes of the midbrain are enriched for *Cd38* expression and that mice lacking *Cd38* show elevated NAD+/NAM ratios in midbrain and striatum. Numerous changes in gene expression were identified in *Cd38* ^*+/−*^ and *Cd38* ^*−/−*^ astrocytes related to the regulation of cellular health, responses to stress, and bioenergetics. *Cd38* ^*−/−*^ astrocytes displayed altered bioenergetic oxygen consumption and glycolysis as compared to *Cd38* ^+/+^ mice. Altogether, these results demonstrate that CD38 is essential for the maintenance of the transcriptional state and bioenergetics in astrocytes. These findings provide the basis for future studies exploring the potential roles for CD38 in astrocyte‐mediated support of neurons with age and disease.

## 2 Methods

### 2.1 Animals

All animals were maintained in accordance with Virginia Tech and the University of Alabama at Birmingham veterinary and IACUC protocols respective to each institution. Mice were housed in facilities on a 12‐h reverse light/dark cycle with ambient temperature and humidity maintained at all times across experiments. Mice had access to purified water and standard chow *ad libitum*. Animal health was monitored daily by veterinary staff with all efforts taken to minimize pain and distress. All mice used were bred from the C57BL/6J strain. *Cd38* knockout mice (B6.129P2‐*Cd38tm1Ln*/J, The Jackson Laboratory) have a global disruption in exons 2–3 of the *Cd38* ^*tm1Lnd*^ allele, resulting in heterozygous mice (*Cd38* ^*+/−*^) with an approximate 50% decrease in CD38 presence and knockout animals (*Cd38* ^*−/−*^) having nearly no *Cd38* transcript and CD38 protein (validated herein and (Cockayne et al. 1998)). All animal comparisons were made against litter and/or peer‐matched controls. Samples were blinded for analysis to reduce potential biases.

### 2.2 Fluorescence In Situ Hybridization

FISH was performed using the RNAscope Multiplex Fluorescent assay (Advanced Cell Diagnostics, Cat. #: 28295574) according to the manufacturer's instructions and as previously described (McMeekin et al. 2018). Mice were briefly anesthetized with isoflurane and rapidly decapitated prior to brain removal. Brains were snap‐frozen over dry ice for cryostat sectioning. Tissue was cut into 20 μm sections and collected on SuperFrost Plus slides (Thermo Fisher Scientific, 1255015) prior to a subsequent freeze. Samples were fixed in 4% ice‐cold paraformaldehyde, followed by dehydration in ethanol and pretreatment in protease IV (Advanced Cell Diagnostics). Tissues and probes (*Cd38*, Cat. #: 513061, *Th*, Cat. #: 317621‐C3, and *Gja1*, Cat. #: 486191‐C2) were incubated at 40°C for 2 h, followed by fluorescent amplification and mounting with Prolong diamond antifade mounting medium containing DAPI (Thermo Fisher Scientific, P36962). Images were acquired by a confocal microscope (Nikon A1+ model, Nikon Inc.).

### 2.3 Reverse‐Transcription Quantitative PCR

RT‐qPCR was conducted as previously described (McMeekin et al. 2018). Briefly, mice were anesthetized with isoflurane and decapitated for rapid removal and dissection of brain tissue. Tissues were flash frozen on dry ice and stored at −80°C. Once removed from storage, tissue was prepared by incubation in RNAlater‐ICE (Ambion) according to the manufacturer's instructions. Tissue was homogenized in TRIzol using a Tissue‐Tearor homogenizer (Biospec). RNA was isolated using the TRIzol/choloform‐isopropanol method following the manufacturer's instructions (Invitrogen). RNA concentration and purity were determined using a NanoDrop 2000 (Thermo Fisher Scientific). Equivalent amounts of RNA (1 μg) were treated with DNase I (Promega) at 37°C for 30 min and DNase Stop solution at 65°C for 15 min. RNA was reverse‐transcribed using the High‐Capacity cDNA Archive Kit (Applied Biosystems). Transcripts were measured using mouse‐specific primers from Applied Biosystems and JumpStart Taq Readymix (Sigma‐Aldrich) using a protocol with an initial ramp (2 min, 50°C; 10 min, 95°C) and 40 subsequent cycles (15 s, 95°C; 1 min, 60°C). Relative concentration of transcript was calculated compared with a standard curve generated from pooled cDNA samples and then diluted (1:5, 1:10, 1:20, 1:40; calibrator method). Obtained values were normalized to β‐actin and expressed as a ratio to control samples ± SEM. The following primer/probe sets were used: *Actb* (Mm00607939_s1, Thermo Fisher Scientific, Cat. #: 4331182) and *Cd38*, (Mm01220904_m1, Thermo Fisher Scientific, Cat. #: 4351372).

### 2.4 Western Blot Analysis

Midbrain tissue was dissected and subsequently homogenized in RIPA buffer (50 mM Tris, 150 mM NaCl, 1% Triton X‐100, 0.5% sodium deoxycholate, 0.1% SDS). A total of 10 μg of protein per sample (Pierce BCA protein assay kit, Thermo Fisher Scientific, Cat. #: 23225) was combined with 4X loading buffer (100 mM Tris, 4% (w/v) SDS, 20% (v/v) glycerol, 0.2% (w/v) Bromophenol blue and 200 mM DTT). The mixture was heated at 65°C for 10 min. Protein samples were then loaded onto a Mini‐PROTEAN TGX gel (4% to 20%, Bio‐Rad) and electrophoresed for 30 min at 200 V. Protein from the gel was transferred to the nitrocellulose membrane (Trans‐Blot Turbo RTA Mini 0.2 μm Nitrocellulose kit, Bio‐Rad) using a Bio‐Rad standard Turboblot transfer system. After transfer, the membrane was washed with 1X TBS. To avoid non‐specific binding, the membrane was blocked using LI‐COR blocking buffer and TBST in a 1:1 dilution before incubating overnight with the rabbit anti‐CD38 (Abcam, Cat. #: ab216343) at 1:1000 and mouse anti‐actin (Millipore Sigma, Cat. #: A5441) at 1:10,000 at 4°C. The immunoblot was washed three times using TBST buffer for 10 min in a shaker, followed by adding anti‐mouse (IRDye 800CW Goat Anti‐Mouse antibody, Cat. #: 926‐32210) and anti‐rabbit (IRDye 680CW Goat Anti‐Rabbit antibody, Cat. #: 925‐68071) secondary antibodies and incubated at room temperature for 1 h. Subsequently, the blot was washed 3X for 10 min using TBST, followed by a 1X TBS wash for 5 min. Amersham ImageQuant 800 biomolecular imager system (Cytiva) was used to capture the image, and band intensity quantification was done using ImageQuant analyzer software (Cytiva). CD38 band intensity was normalized to actin.

### 2.5 ^1^H NMR Analysis

Striatum and midbrain samples from *Cd38* ^*+/+*^, *Cd38* ^*+/−*^, and *Cd38* ^*−/−*^ mice were microdissected then pooled into 1.5 mL microcentrifuge tubes, snap frozen in liquid nitrogen, and stored at −80°C. Tissues were placed in 15 mL tubes and 4 mL of 80% ice‐cold methanol per gram of tissue was added. Tissues were placed in 1.5 mL microcentrifuge tubes, and 80% ice‐cold methanol was added at a ratio of 4 μL per milligram of tissue. The tissues were homogenized using homogenizer pestles, followed by grinding for 30 s. Chloroform was then added to each sample at a ratio of 2 μL per milligram of tissue, followed by an additional 30 s of homogenization. Next, chloroform and HPLC‐grade water were added to each sample at a ratio of 2 μL each per milligram of tissue. The samples were incubated on ice for 30 min and then centrifuged at 4000 g at 4°C for 30 min. The upper aqueous layer of the supernatant, containing the metabolites, was extracted for NMR analysis. Samples were dried using a heat block at 42°C followed by lyophilization using a freeze‐dry system. Samples were then dissolved in 0.6 mL D_2_O (99.96% D) and transferred to NMR tubes. Tetramethylsilane (0 ppm) was added as a reference.

Experiments were conducted at 25°C using a Bruker Avance III‐HD spectrometer (^1^H, 600 or 850 MHz) equipped with cryogenic triple‐resonance probes. Data were processed and analyzed with TopSpin (v. 4.2). ^1^H spectra were recorded with 32 k complex points, an acquisition time of 1.6 s, a recovery delay of 2.0 s, and pre‐saturation of the residual HDO signal. A 0.5 Hz exponential apodization was applied and data size doubled by zero‐filling before Fourier transformation. Each spectrum was phased manually, and automatic baseline correction was achieved using a 2nd order polynomial in the region 0.5–9.5 ppm. Integral intensities were scaled identically for all spectra within each series. Final integration values for metabolites were the average of integration values obtained for a set of five samples.

### 2.6 Magnetic Bead Astrocyte Isolation

Astrocytes were isolated from *Cd38* ^*+/+*^, *Cd38* ^*+/−*^, and *Cd38* ^*−/−*^ male mice after 1 year of age (12–14 months, *x̄* = 13.5 months) utilizing a MACS approach (Holt and Olsen 2016; Holt et al. 2019). Midbrain regions associated with Parkinson's disease pathology (striatum, thalamus, and substantia nigra) were microdissected from brain tissue for cell‐specific isolation. Tissue dissociation was performed as previously described (Holt and Olsen 2016; Holt et al. 2019), whereby regions of interest were utilized for MACS. All dissections were performed in modified artificial cerebral spinal fluid (120 mM NaCl, 3 mM KCl, 6 mM 2NaHCO_3_, 11 mM glucose, 5 mM HEPES, 200 μM CaCl_2_, 1 mM MgCl_2_) containing neuronal activity inhibitors (20 μM CNQX, 20 μM D‐AP5, 295–299 mOsm) to reduce glutamate excitotoxicity. Briefly, midbrain tissues were removed then sectioned into 1 mm pieces, followed by papain dissociation (Worthington Biochemical Corporation, Cat. #: LK003150). A series of centrifugation (4°C, 300 RCF, 3–5 min, Eppendorf, Cat. #: 58048R) and PBS wash steps were performed to retain cell‐containing tissue pellets. Supernatants were discarded and pellets were resuspended in 0.5% w/v bovine serum albumin (Millipore Sigma, Cat. #: A7030) in PBS for filtration (70 μm, Fisher Scientific, Cat. #: 22‐363‐548) and attainment of midbrain cellular fractions. Cell fractions were then treated with Miltenyi Biotec Microbeads to remove contaminating endothelial cells (Cat. #: 130‐097‐418) and myelin/oligodendrocytes (Cat. #: 130‐096‐433). Samples were incubated in microbeads for 10 min at 4°C followed by filtering through Miltenyi LS columns (Cat. #: 130‐042‐401). Samples were then treated with anti‐CD11b microbeads (Cat. #: 130‐092‐263) for 10 min at 4°C to remove microglia by elution of magnetically captured cells. Finally, astrocytes were positively selected using the Miltenyi ACSA‐II microbead kit (Cat. #: 130‐097‐679). Samples were first incubated with an FcR blocking reagent for 10 min at 4°C immediately followed by an additional 10‐ min incubation at 4°C with anti‐ACSA‐2 microbeads. After LS column filtration and elution, collected cells were stored in 300 μL Trizol (Thermo Fisher Scientific, Cat. #: 15596026) and snap frozen before being placed in storage at −80°C. Cellular fraction mRNA was isolated for qPCR and prepared as described (2.3 Reverse‐transcription quantitative PCR). The following Taqman primer/probe sets were purchased from Thermo Fisher Scientific: microglia‐ #Mm00525305_m1 *Tmem119*, oligodendrocytes‐ #Mm01266402_m1 *Mbp*, astrocytes‐ #Mm00439105_m1 *Gja1*, Cd38‐ #Mm01220904_m1, dopaminergic neurons‐ #Mm00447557_m1 *Th* and #Mm01353211_m1 *Drd1*. The Mouse GAPDH Control DQ mix (Applied Biosystems, Cat. #: 4351309). Relative expression of transcript was calculated using the 2^−ΔΔCt^ method, and relative expression normalized to whole midbrain expression for each probe.

### 2.7 RNA‐Sequencing

Isolated midbrain astrocytes were gently lysed by syringe for preparation of RNA isolation (Zymo Research, Cat. #: R2062). RNA was extracted according to the Direct‐zol RNA Microprep protocols, including DNA degradation with DNase I. Total RNA was eluted in 10 μL of DNase/RNase free water. Samples were submitted to Medgenome for rRNA depletion, quality verifications, library construction, and ultra‐low input RNA sequencing. RNA integrity was determined by Qubit (Thermo Fisher Scientific) fluorometric quantitation and Tapestation Bioanalyzer (Agilent) analysis. Collected RNA was then converted to cDNA samples by using the Takara SMART‐Seq v4Ultra low Input RNA kit. Generated cDNA was checked for quality utilizing Qubit and Tapestation measures, whereby libraries with marginal pass and higher quality were used for 100 bp paired‐end sequencing on the NovaSeq 6000 (Illumina) platform.

### 2.8 Sequencing Analysis

After RNA‐seq data acquisition, bases with quality scores less than 30 were removed and adapters were trimmed from raw sequencing reads by Trim Galore (v 0.6.4). After trimming, only reads with length greater than 30 bp were mapped to mm10 by STAR (v 2.7.1a). Raw counts and normalized counts for each gene were output by RSEM (v1.2.28). Gene expression in TPMs for each library can be found in Table S1. Genes with an average TPM greater than 5 in at least one group were used to identify differentially expressed genes (DEGs). Only genes with an adjusted *q*‐value less than 0.05 and at least 1.2‐fold change (FC) were considered as DEGs. All DEG pairwise comparisons can be found in Table S2. DEGs were used for GO analysis performed with ClueGO (v2.5.10) (Bindea et al. 2009). GO analysis results can be found in Table S3. DEGs were further evaluated relative to available datasets representing PD risk association studies (Kia et al. 2021; Yao et al. 2021; Zhou et al. 2023), genes enriched in midbrain astrocytes (Saunders et al. 2018), common genes activated in reactive astrocytes (Hasel et al. 2021), age‐related striatal astrocyte genes (Clarke et al. 2018), genes involved in reactive oxygen species (ROS) pathways (Rouillard et al. 2016), and mitochondrial function genes (Rath et al. 2021).

### 2.9 Metabolic Assays

Functional metabolic in situ assays were performed on late‐adulthood aged *Cd38* ^*+/+*^, *Cd38* ^*+/−*^, and *Cd38* ^*−/−*^ mice from a convenience sample of male and female basal ganglia (8–12 months, *x̄* = 10.34 months). Midbrain tissue was removed via microdissection and snap‐frozen for protein‐based applications. Samples were brought to the Virginia Tech Metabolism Core for functional measures of mitochondrial bioenergetics. Measures of pyruvate dehydrogenase, citrate synthase, and cytochrome *c* oxidase enzymatic activity were assayed (see below). Midbrain samples were homogenized in SET buffer (250 mM sucrose, 1 mM EDTA, 10 mM Tris–HCl, and 1 mM ATP, pH 7.4) then distributed across assays. Data were normalized to total protein content per sample.

Mitochondrial electron transport chain function was evaluated by measuring the oxidation rate of cytochrome *c* oxidase. Homogenized samples were added to a solution containing 2 mg/mL reduced cytochrome c reduced with 10 mg/mL sodium dithionite dissolved in a 0.1 M potassium phosphate buffer that contained 1 mg/mL of BSA and 120 mM of lauryl maltoside. Absorbance measures (550 nm) were taken every 10 s for 5 min to measure the oxidation rate of reduced cytochrome *c*. Potassium cyanide (240 μM) was used to inhibit the reaction to ensure the slope was specific to COX. Triplicates of each reading were taken for each sample.

Citrate synthase function was determined by measuring the reduction of DTNB, a process downstream of citrate synthase's catalyzation of citrate and CoASH from acetyl‐CoA and oxaloacetate. Homogenized midbrain samples were added in duplicate to 170 μL solution containing Tris buffer (0.1 M, pH 8.3), DTNB (1 mM, in 0.1 M in Tris buffer), and oxaloacetate (0.01 M, in 0.1 M Tris buffer) at a 1:5 ratio of sample to buffer, respectively. Spectrophotometer (SPECTRAmax ME, Molecular Devices Corporation, Sunnyvale, California) readings were taken after background subtraction. To initiate the reaction, 30 μL of 3 mM acetyl‐CoA per sample was kept at 37°C. Absorbance (405 nm) was measured every 12 s for a total of 7 min. Triplicates of each reading were taken for each sample.

### 2.10 Human Data Curation

Previous works have identified significant associations between the *BST1/FAM200B/CD38* regions, localized to chromosome 4 (Kent et al. 2002), and PD risk (Chang et al. 2017; Nalls et al. 2019). Relative expression profiles for *BST1*, *FAM200B*, and *CD38* mRNA in human tissues were explored by genotype at that locus using GTExPortal version 8 (GTEx Consortium 2020). Cell type‐specific expression data for human cortex was explored using the Human Protein Atlas database single cell database (Karlsson et al. 2021), the Allen Brain Atlas, (Allen Brain Atlas 2006) and the Oldham Brain Expression Correlates database (Kelley et al. 2018). Cell type and region‐specific transcriptional data for mice was downloaded from Dropviz.org; data were normalized to the total unique molecular identifier (UMI) hits for each cell subcluster and multiplied by 100,000, similar to the browser tool. Cell types were categorized based on criteria described by others (Saunders et al. 2018). For the compilation of genes implicated by PD genetic studies, we compiled risk SNPs from recent GWAS studies and used GTExPortal to identify genes affected at the expression or splicing level in human tissues (Chang et al. 2017; Nalls et al. 2014).

### 2.11 Immunofluorescence Analysis

Substantia nigra sections of 40 μm were obtained from perfused, one‐year‐old *Cd38* ^*+/+*^ and *Cd38* ^*−/−*^ mice for immunofluorescence analysis of markers for astrocytic reactivity (GFAP) and dopaminergic neurons (TH). Briefly, nigra sections were incubated in a 10% pre‐incubation normal donkey serum blocking buffer for 1 h at RT followed by four, 5‐min washes in 0.3% PBS‐Tween (1X PBS, Gibco, Ref # 10010‐031, and Tween 20, Millipore Sigma, CAS‐No 9005‐64‐5). Sections were then incubated overnight with primary antibodies for mouse anti‐GFAP (Cell Signaling Technology, Cat. #: 3670S) at 1:500, and chicken anti‐Tyrosine Hydroxylase (Abcam, Cat. #: ab76442) at 1:500. Sections were washed in 0.3% PBS‐Tween with four, 5‐min washes. Secondary antibodies for anti‐mouse (Alexa Fluor 555, Donkey Anti‐Mouse, Life Technologies, Cat. #: A31570) at 1:1000, and anti‐chicken (Alexa Fluor 680, Donkey Anti‐Chicken, Jackson ImmunoResearch, Code. #: 703‐625‐155) at 1:1000 were incubated with the nigra sections for 2 h. Subsequently, sections were washed with four, 5‐min washes in 0.3% PBS‐Tween, followed by a 5‐min incubation in DAPI (Invitrogen, Cat. #: 62248). Following this, sections were washed with four, 5‐min washes in 0.3% PBS‐Tween and incubated in an autofluorescence eliminator reagent (Millipore Sigma, Cat. #: 2160) for 5 min. Sections were washed with four, 5‐min washes in 0.3% PBS‐Tween and transferred to 1X PBS (Gibco, Ref # 10010‐031) for mounting. Sections were mounted on Superfrost Plus Slides (Epredia, Ref # 4951PLUS‐001) in Prolong Diamond Antifade Mountant (Invitrogen, Ref # P36961) and left to dry in the dark room overnight before confocal imaging on a Nikon TiX2. Images were captured at a 2048 scan area with an averaging of 4, and a pinhole of 1 AU. For the channels, the following settings were used: 405 (Laser: 45.1, Gain: 66), 488 (Laser: 3.5, Gain: 18.5), 561 (Laser: 14, Gain: 38.6), and 640 (Laser: 33.7, Gain: 58.7). Using ImageJ (FIJI), the substantia nigra pars compacta (SNc) and reticulata (SNr) regions were defined using the RGB image and guided by TH neuron localization. Using the predetermined SNc and SNr ROIs, three 250 × 250‐pixel boxes were selected using the TH neurons as the guiding image. Those ROIs were overlaid onto 561‐channel images for mean intensity measurements of GFAP. Before measurements, 2048 × 2048 RGB images were converted to 8‐bit.

For the quantification of SOX9 positive nuclei in the SNc, 50 μm sections of one‐year‐old *Cd38* ^*+/+*^ and *Cd38* ^*−/−*^ mice were stained using the similar technique mentioned above using SOX9 antibody (Abcam, Cat. #: 185966) as an astrocyte nuclear marker and TH (Abcam, Cat. #: ab76442) as a dopaminergic neuronal marker. Nikon A1 confocal was used to image the 50 μm z‐stack images with 0.1 μm steps. After that, ITCN (Automated nuclei counter plugin for ImageJ) Plugin for ImageJ was used to count the SOX9 positive nuclei in the SNc area by considering the TH marker as the guiding area for dopaminergic neurons.

## 3 Results

### 3.1 *Cd38* Is Enriched in Substantia Nigra Astrocytes and Is Required for NAD/NAM Balance and Metabolic Homeostasis in the Midbrain

**FIGURE 1:**
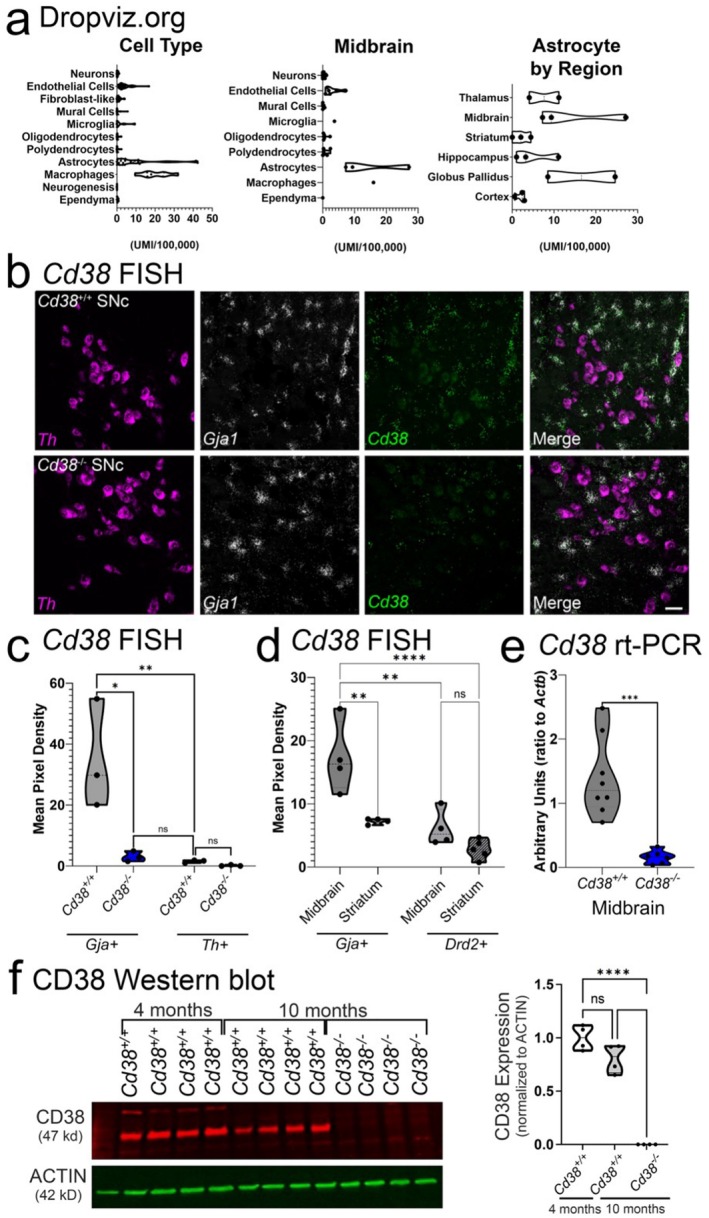
*Cd38* mRNA is primarily expressed by astrocytes in mouse brain. (a) DropViz (Saunders et al. 2018) meta‐analysis reveals that astrocytes have high *Cd38* abundance relative to other cell types (left), especially so in midbrain regions (middle). Basal ganglia astrocyte populations show high abundance (right). (b) Representative FISH images of *Cd38* ^*+/+*^ (top) and *Cd38* ^*−/−*^ (bottom) for tyrosine hydroxylase (*Th*), connexin‐43 (*Gja1*), and *Cd38* mRNA in the midbrain. (c) Single‐cell quantification of *Cd38* in *Gja1*‐positive or *Th*‐positive cells in *Cd38* ^*+/+*^ and *Cd38* ^*−/−*^ mice. 2‐way ANOVA with Tukey's post hoc test, *p < 0.05, **p < 0.01. Scale bar = 25 μm. (d) Single‐cell quantification of *Cd38* mRNA in *Gja1*‐positive cells of *Cd38* ^*+/+*^ mice in comparison to *Drd2+* neurons (dopaminergic neurons in midbrain; spiny projection neurons in dorsolateral striatum). 2‐way ANOVA with Tukey's post hoc *t‐*test, **p < 0.01; ****p < 0.0001. (e) RT‐qPCR quantification of *Cd38* mRNA in midbrain from *Cd38* ^*+/+*^ and *Cd38* ^*−/−*^ mice. Unpaired *t*‐test assuming unequal variances. ***p < 0.001. (f) CD38 protein was detected in midbrain homogenates of *Cd38* ^*+/+*^ mice (4 or 10 months of age) or *Cd38* ^*−/−*^ mice (10 months). One‐way ANOVA, Tukey's post hoc *t*‐test, ****p < 0.0001.

While some publications suggest that *Cd38* mRNA expression is present across multiple brain cell types (for review, see (Guerreiro et al. 2020)), we sought to empirically determine cell type‐specific expression in mouse brain with publicly available datasets and small molecule fluorescent in situ hybridization (FISH). First, we explored the expression of *Cd38* mRNA by region and cell type in mouse brain using Dropviz.org (Saunders et al. 2018), with empirical evaluation of gene expression patterns using FISH in wildtype (*Cd38* ^*+/+*^) and *Cd38* knockout (*Cd38* ^*−/−*^) brain tissues. These analyses revealed *Cd38* is most abundant in astrocytes and brain‐derived macrophages, with very low expression in neurons of any type (Figure 1a). Using FISH, *Cd38* mRNA expression was localized to astrocytes positive for the expression of *Gja1*, the astrocyte‐enriched gene encoding connexin 34, with little localization of transcript to dopaminergic neurons expressing transcript for tyrosine hydroxylase (*Th*; Figure 1b). Quantification of transcript abundance per cell revealed higher abundance of *Cd38* mRNA in astrocytes than dopaminergic neurons of *Cd38* ^*+/+*^ mice, with almost complete ablation of *Cd38* mRNA in *Cd38* ^*−/−*^ mice (Figure 1c). In support of Dropviz.org data, *Cd38* was found to be more abundant in astrocytes of the midbrain than astrocytes of the dorsolateral striatum (Figure 1d), when compared in sagittal sections containing both substantia nigra and striatum, with *Drd2* used to identify regions with spiny projection neurons and dopaminergic neurons, respectively. Assessment of *Cd38* mRNA abundance in midbrain homogenates confirmed almost complete deletion of transcript in *Cd38* ^*−/−*^mice (Figure 1e), and ablation of CD38 protein was confirmed with Western blotting (Figure 1f). Interestingly, while previous work has documented an increase in CD38 expression with age, this was not observed between 4 and 10 months of age in the midbrain (Figure 1f). Altogether, these data suggest that, in the vicinity of dopaminergic neurons, *Cd38* mRNA is enriched in astrocytes, mRNA and protein are ablated in the brains of *Cd38* ^*−/−*^ mice, and the FISH probe and antibody are specific for CD38 transcript and protein.

**FIGURE 2:**
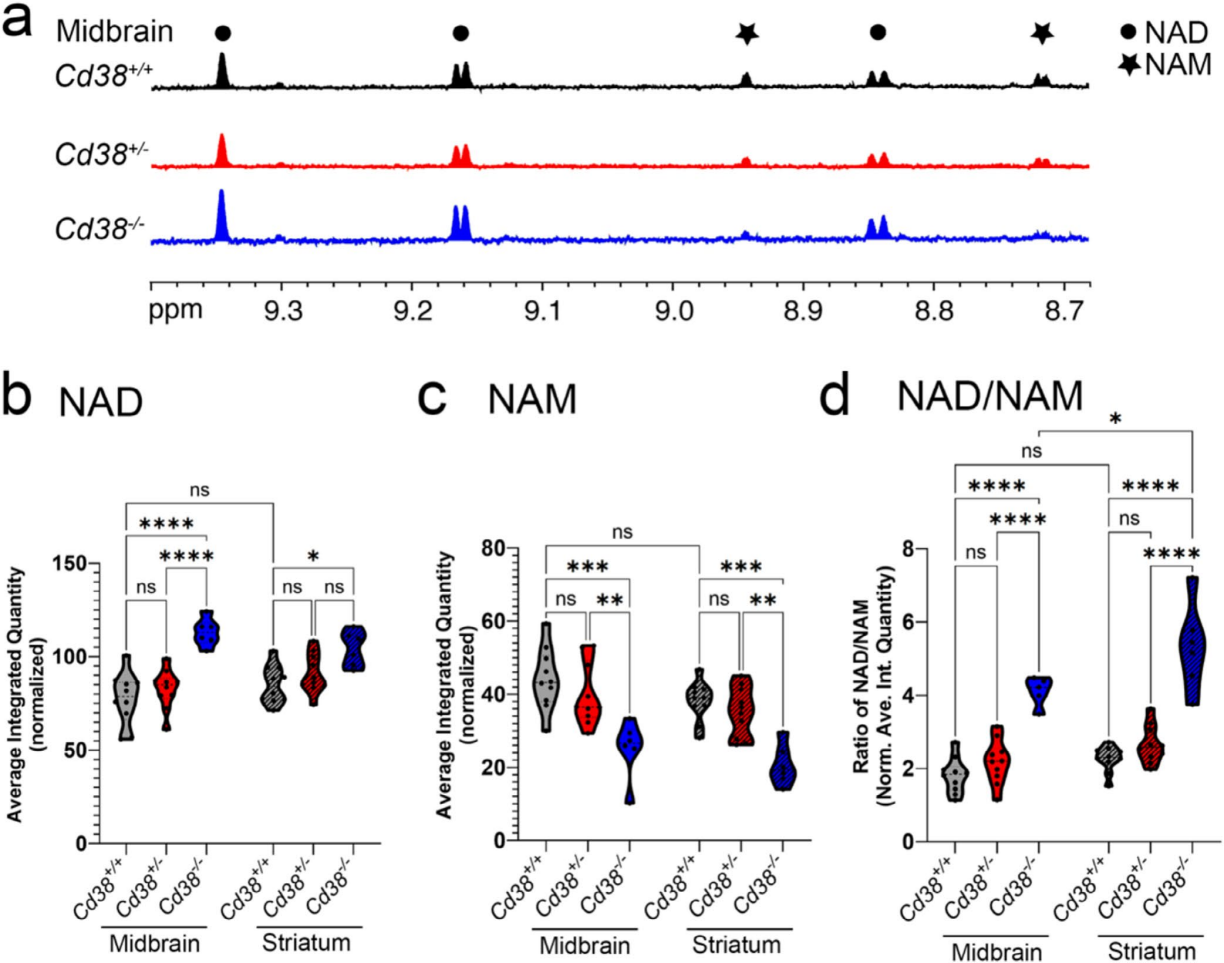
NAD+/NAM ratio changes in the brain of *Cd38*‐deficient mice. (a) ^1^H NMR spectra of three representative midbrain tissue samples showing NAD+ and NAM peaks. Plots showing quantifications of NAD+ (b) and NAM (c) as average integrals of the ^1^H NMR signals and the ratio of NAD+ to NAM (d). Two‐way ANOVA with Tukey's multiple comparisons post hoc *t*‐test *p < 0.05, **p < 0.01, ***p < 0.001, ****p < 0.0001.

To determine whether NAD+ to NAM conversion is dependent on CD38 in the brain, similar to peripheral tissues (Aksoy et al. 2006; Braidy et al. 2014), we next assessed whether *Cd38* ablation had an impact on NAD and NAM levels in the midbrain. Here, we used nuclear magnetic resonance (^1^H NMR) to quantify NAD and NAM in tissue homogenates of midbrain with comparison to the striatum from mice 10–12 months of age (representative traces from midbrain data shown in Figure 2a). Complete deletion of *Cd38* caused an increase in NAD+ tissue concentrations in both brain regions (Figure 2b), with a decrease in NAM (Figure 2c) and a corresponding increase in the NAD+/NAM ratio (Figure 2d). Mice heterozygous for the *Cd38* mutant allele did not show any changes in NAD+, NAM, or NAD+/NAM ratio in either brain region.

### 3.2 RNA‐Sequencing Reveals Changes in Transcript Abundance for Metabolic, ROS, and Age‐Related Genes in *Cd38^+/−^* and *Cd38* ^*−/−*^ Astrocytes

**FIGURE 3:**
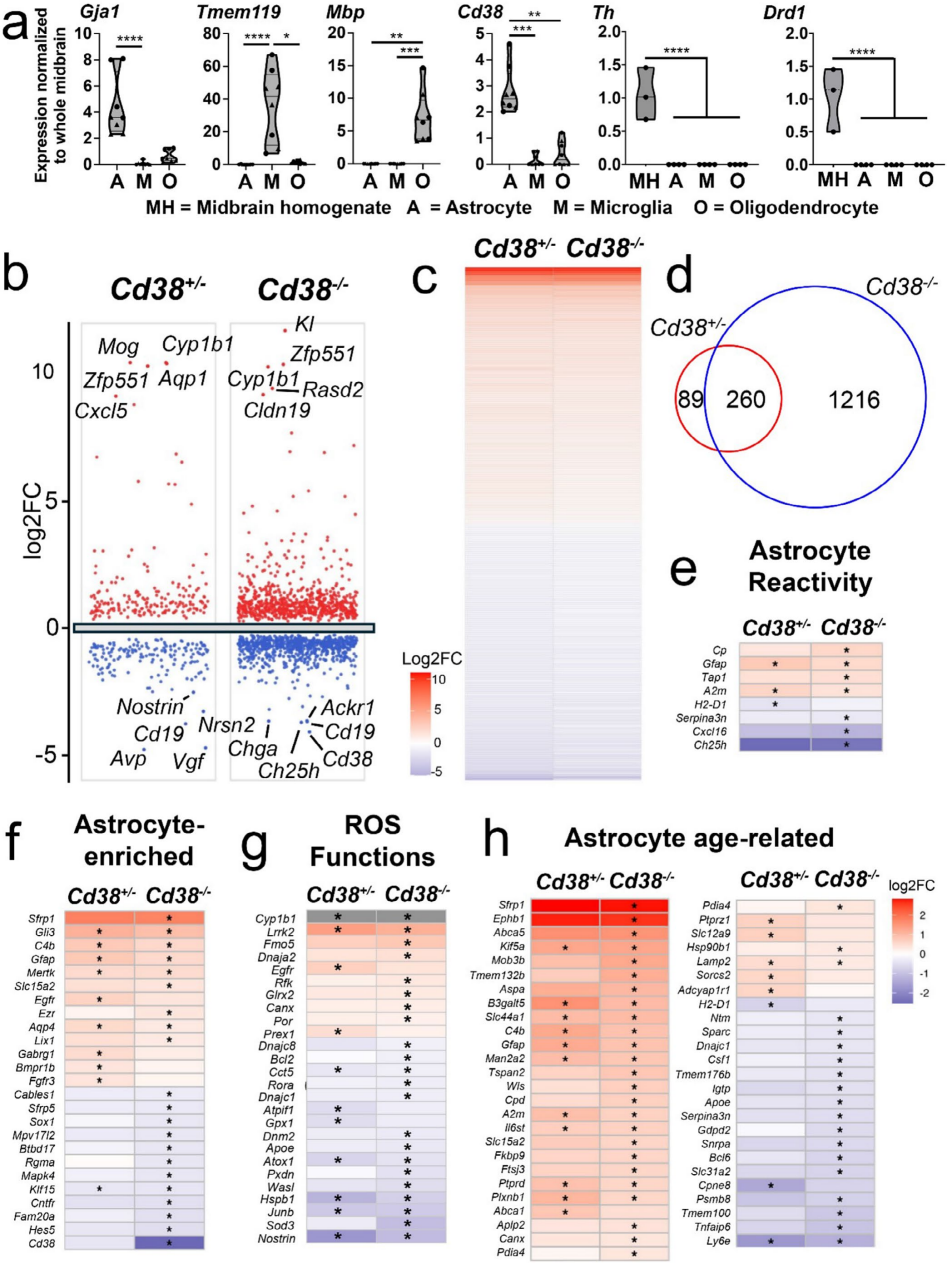
*Cd38*‐deficient astrocytes show robust transcriptional changes. (a) Quantitative PCR of astrocytes, microglia and oligodendrocytes isolated from male (circles) and female (triangle) midbrain confirms purity of pull‐downs and *Cd38* enrichment in astrocytes. Kruskal‐Wallis, Dunn's multiple comparisons test *p < 0.05, **p < 0.01, ***p < 0.001, ****p < 0.0001. (b) Volcano plots from *Cd38* ^*+/−*^ vs. *Cd38* ^*+/+*^ astrocyte comparisons and *Cd38* ^*−/−*^ vs. *Cd38* ^*+/+*^ comparisons show all upregulated (red), downregulated (blue) differentially expressed genes (DEGs). The 5 most highly upregulated and downregulated significant DEGs by fold‐change (log2FC) are labeled. (c) Heatmap indicating 260 shared DEGs in (d) change their expression in the same direction. (d) Venn diagram indicating unique and shared DEGs from *Cd38* ^*+/−*^ and *Cd38* ^*−/−*^ astrocytes, compared to *Cd38* ^*+/+*^ astrocytes. Heatmaps for comparisons against available datasets of genes associated with astrocyte reactivity (e), astrocyte‐enrichment (f), ROS function (g), and age (h). a, *n* = 4 male and 4 female mice, b–d, *n* = 3, *Cd38* ^*+/+*^, *n* = 3 *Cd38* ^*+/−*^, *n* = 6, *Cd38* ^*−/−*^, (*q* ≤ 0.05, log2FC > 1.2). For Figure 3g, gray box represents > 10‐fold change.

Considering the enrichment of *Cd38* in astrocytes, we then assessed the effects of *Cd38* deficiency on the metabolic state of astrocytes using RNA sequencing and functional assays. Cell‐specific MACS isolation protocols (Holt and Olsen 2016; Holt et al. 2019) were utilized to separate astrocytes for RNA sequencing. First, we validated this approach in the midbrain from mice (Figure 3a), demonstrating the enrichment of the astrocyte marker gene *Gja1* in populations pulled down with ACSA‐2 (astrocyte cell surface antigen 2) antibody‐bound beads, enrichment of the microglial marker gene *Tmem119* in populations pulled down with CD11b‐antibody‐bound beads, and the oligodendrocyte marker gene *Mbp* in the remaining cells. Importantly, preferential expression of *Cd38* by astrocytes relative to the other glial populations was confirmed in this pull‐down (Figure 3a). Additionally, qPCR for the neuronal markers tyrosine hydroxylase (*Th*) and the dopamine receptor D1 (*Drd1*) revealed no neuronal contamination in astrocytes isolated from the midbrain (Figure 3a). Based on these results we next isolated midbrain astrocytes from aged (12–14 months, *x̄* = 13.5 months) *Cd38* ^*+/+*^, *Cd38* ^*+/−*^, *and Cd38* ^*−/−*^ mice for next‐generation RNA‐seq applications. Pairwise comparisons between *Cd38* ^*+/−*^ to *Cd38* ^*+/+*^ and *Cd38* ^*−/−*^ to *Cd38* ^*+/+*^ were performed to identify DEGs (Table S2), followed by a GO pathway analysis (Table S3).

We were able to identify 349 significant DEGs in *Cd38* ^*+/−*^ to *Cd38* ^*+/+*^ comparisons, and a total of 1476 significant DEGs were identified in *Cd38* ^*−/−*^ to *Cd38* ^*+/+*^ comparisons (Figure 3b, *Cd38* ^*+/−*^ vs. *Cd38* ^*+/+*^: 183 upregulated and 166 downregulated; *Cd38* ^*−/−*^ vs. *Cd38* ^*+/+*^: 718 upregulated and 762 downregulated). Of these, 89 DEGs were unique to the *Cd38* ^*+/−*^ to *Cd38* ^*+/+*^ comparison, 260 DEGs were shared between the *Cd38* ^*+/−*^ to *Cd38* ^*+/+*^ and *Cd38* ^*−/−*^ to *Cd38* ^*+/+*^ comparisons, and 1216 DEGs were unique to *Cd38* ^*−/−*^ to the *Cd38+/+* comparison (Figure 3c,d). Therefore, approximately 75% of DEGs identified in *Cd38* ^*+/−*^ were also identified in *Cd38* ^*−/−*^ comparisons, while the unique set of *Cd38* ^*−/−*^ DEGs compromised approximately 82% of identified genes (Figure 3d).

Overlapping DEGs revealed differentially expressed genes involved in astrocyte activation (in the context of LPS‐mediated inflammation (Hasel et al. 2021)) (Figure 3e), genes that are unique to astrocytes or astrocyte‐enriched (Figure 3f), genes involved in reactive oxygen species (ROS) control (Rouillard et al. 2016) (Figure 3g), and genes which have been shown to change in astrocytes with age (Clarke et al. 2018) (Figure 3h). Regulation of ROS is paramount as free radicals can intercept electrons and damage cellular DNA/RNA, proteins, and membranes. Additionally, increased cellular ROS accumulation incurs mitochondrial damage and can contribute to bioenergetic dysfunction (Nissanka and Moraes 2018). Upregulated genes of interest include those that regulate water homeostasis (*Aqp4*) and inflammatory status (*Gfap, C4b, Cxcl5*). *Gfap* is of particular interest as its upregulation suggests elevation in reactive astrogliosis (Lawrence et al. 2023). Astrocytes are also capable of responding to immune system signals and responding as immune effectors (Dong and Benveniste 2001). Relevant to the hallmarks of neurodegenerative disease, *Cd38*‐deficient astrocytes also had disruptions to genes that regulate DNA/RNA functions (*Trex1*), protein homeostasis (*Ubb*), and cellular bioenergetics (*Cox10*). Dysregulation of groups of genes that regulate RhoGTPase activity (*Arhgap27*, *Arhgdia, Arhgef16*), Krüppel‐like factors (*Klf2, Klf6*), solute carriers (*Slc12a6, Slc38a5*), transmembrane proteins (*Tmem161b, Tmem204*), and zinc finger proteins (*Zfp251, Zfp407, Zfp551*) are amongst the many DEGs identified within the overlapping dataset. It is important to note, that within the overlapped DEGs group, directionality was shared between *Cd38* ^*+/−*^ and *Cd38* ^*+/+*^ and *Cd38* ^*−/−*^ and *Cd38* ^*+/+*^ pairwise comparisons. Altogether, dysregulation of essential biological processes is suggested by *Cd38* deficiency.

**FIGURE 4:**
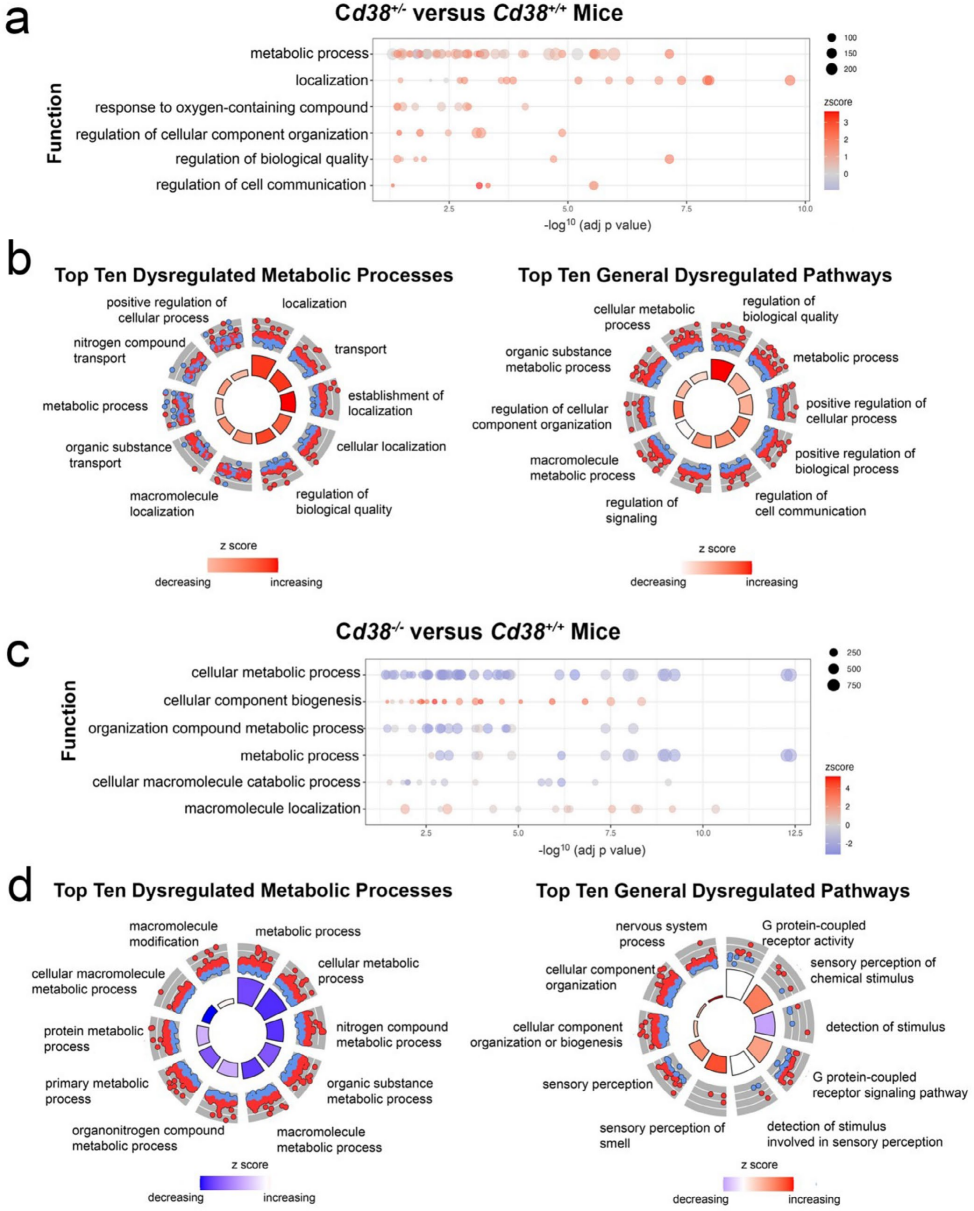
Transcriptomic pathway analysis reveals key differences between control and *Cd38*‐deficient astrocytes. (a) CLUGO GO analysis showing functional groups with the largest number of GO terms comparing *Cd38* ^*+/−*^ vs. *Cd38* ^*+/+*^ astrocytes. Each bubble represents a GO sub‐term associated with the indicated process. Colors represent Z‐scores. The size of the bubbles corresponds to the number of genes in each term. (b) GO CIRCO plot shows the top 10 gene ontology enrichment terms related to metabolic processes comparing *Cd38* ^*+/−*^ vs. *Cd38* ^*+/+*^ astrocytes (left), and the top 10 most significant GO terms in *Cd38* ^*+/−*^ vs. *Cd38* ^*+/+*^ astrocytes (right). (c) CLUGO GO analysis shows functional groups with the largest number of GO terms comparing *Cd38* ^*−/−*^ vs. *Cd38* ^*+/+*^. (d) GO circo plot for the top 10 gene ontology enrichment terms related to metabolic processes comparing *Cd38* ^*−/−*^ vs. *Cd38* ^*+/+*^ (left) and the top 10 most significant GO terms *Cd38* ^*−/−*^ vs. *Cd38* ^*+/+*^ (right).

Following DEG identification, we performed a GO pathway analysis to evaluate dysregulated biological processes as a result of *Cd38* deficiency (Figure 4). These analyses indicated that metabolic processes were over‐represented in the data GO terms when comparing *Cd38* ^+/+^ to *Cd38* ^+/−^ astrocytes, and, indeed, represented 54% of all significantly dysregulated GO terms in this comparison (Figure 4a). GO circo plots show the top 10 gene ontology enrichment terms related to metabolic processes comparing *Cd38* ^*+/−*^ vs. Cd38^+/+^ (Figure 4b) and the top 10 most significant (based on *p* value) dysregulated GO terms, across all functional groups (Figure 4b).

GO pathway analysis of DEGs identified in the *Cd38* ^*−/−*^ to *Cd38* ^*+/+*^ comparison was also performed utilizing the same criteria as above. Utilizing these thresholds, we revealed 173 total significant GO terms. Here, terms identified from total DEGs analyses again identified overrepresentation of terms related to metabolic processes (Figure 4c; 27% of total terms, Table S3). GO circo plots show the top 10 gene ontology enrichment terms related to metabolic processes comparing *Cd38* ^*−/−*^ vs. *Cd38* ^*+/+*^ (Figure 4d, left) and the top 10 most significant (based on *p* value) dysregulated GO terms across all functional groups (Figure 4d, right).

### 3.3 *Cd38* Deficiency Affects Bioenergetics

**FIGURE 5:**
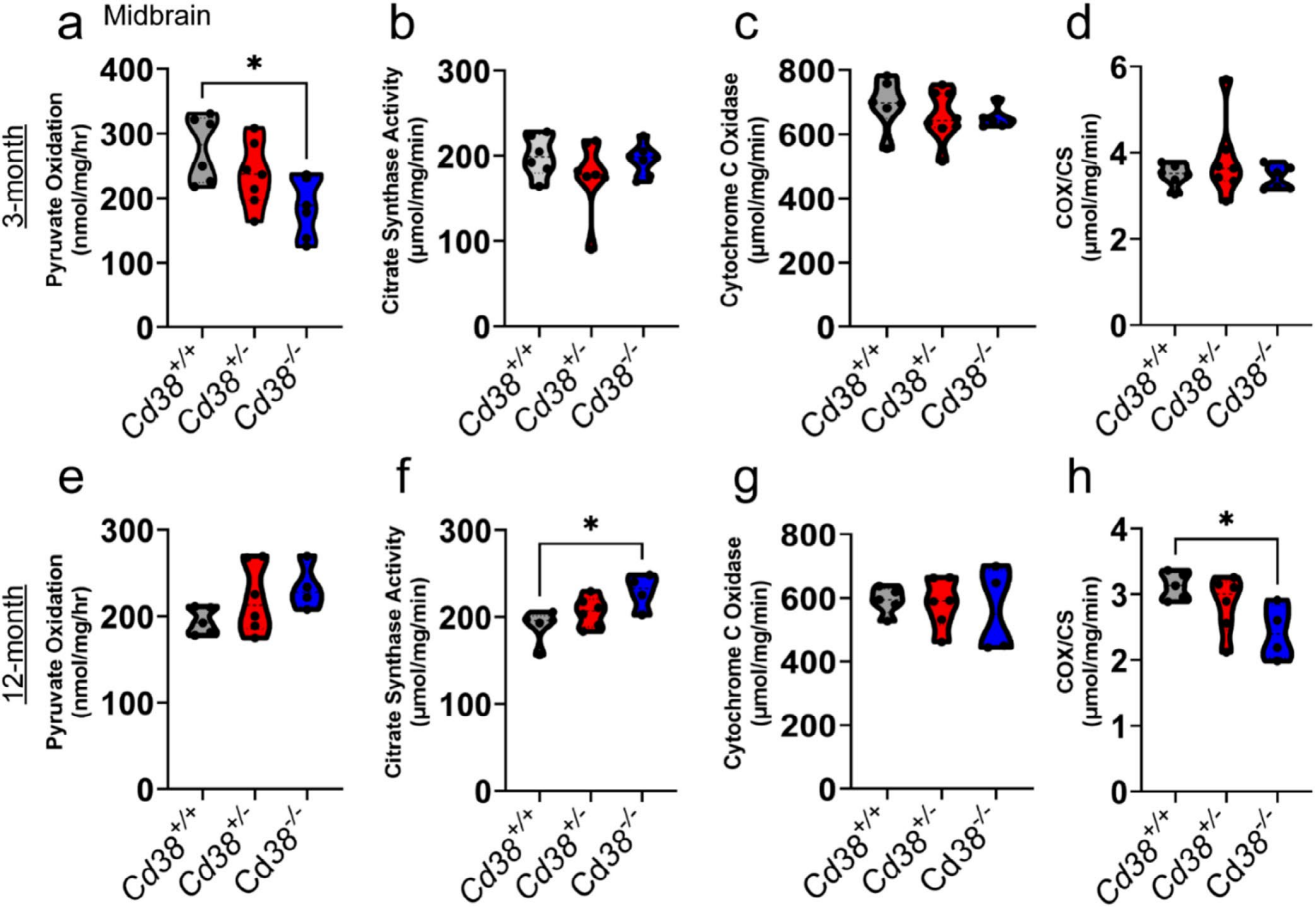
*Cd38* deficiency alters regional bioenergetics. Functional metabolic in situ measurements were assessed in midbrain of 3‐month and 12‐month for pyruvate oxidation (a, e), citrate synthase (CS) activity (b, f), and cytochrome c oxidase (COX) activity (c, g), with normalization of COX activity to CS activity (d, h). One‐way ANOVA, Tukey's multiple comparisons post hoc *t*‐test *p < 0.05. *n* = 4–6/group.

Based on the evidence of metabolic disruptions and the transcriptional changes of metabolic genes in the *Cd38*‐deficient mice, we next explored whether *Cd38* deficiency causes metabolic changes in midbrain tissues of *Cd38* ^*+/+*^, *Cd38* ^*+/−*^, and *Cd38* ^*−/−*^ mice 10–12 months of age. Measures of pyruvate oxidation, citrate synthase (CS), and cytochrome *c* oxidase activity (complex IV) were of interest given the consumption of NAD+ by pyruvate oxidation and TCA cycle activity and the generation of NAD+ by oxidative phosphorylation. In a 3‐month‐old midbrain sample, pyruvate oxidation was significantly downregulated in *Cd38* ^*−/−*^ mice (Figure 5a), while no changes in citrate synthase activity, cytochrome c oxidase and COX/CS ratio (Figure 5b–d) were observed amongst genotypes, suggesting early metabolic dysfunction in *Cd38*‐deficient mice, particularly changes in mitochondrial utilization of pyruvate. In the 12‐month‐old sample, while no statistically significant differences were observed in pyruvate oxidation (Figure 5e), an increase was observed in citrate synthase activity when comparing *Cd38* ^*−/−*^ to *Cd38* ^*+/+*^ midbrain tissues (Figure 5f). We observed no significant differences in cytochrome *c* oxidase between genotypes normalized to protein (Figure 5g), yet COX activity as a ratio to citrate synthase activity was reduced in *Cd38* ^*−/−*^ as compared to *Cd38* ^*+/+*^ mice (Figure 5h), indicating age‐dependent changes in mitochondrial content and dysregulation of oxidative phosphorylation (Complex IV) in the absence of *Cd38*, confirming the metabolic and bioenergetic changes observed in the transcriptional dataset.

### 3.4 Evidence for Increased Glial Reactivity in the Substantia Nigra Pars Compacta (SNc) of *Cd38* ^*−/−*^ Mice

**FIGURE 6:**
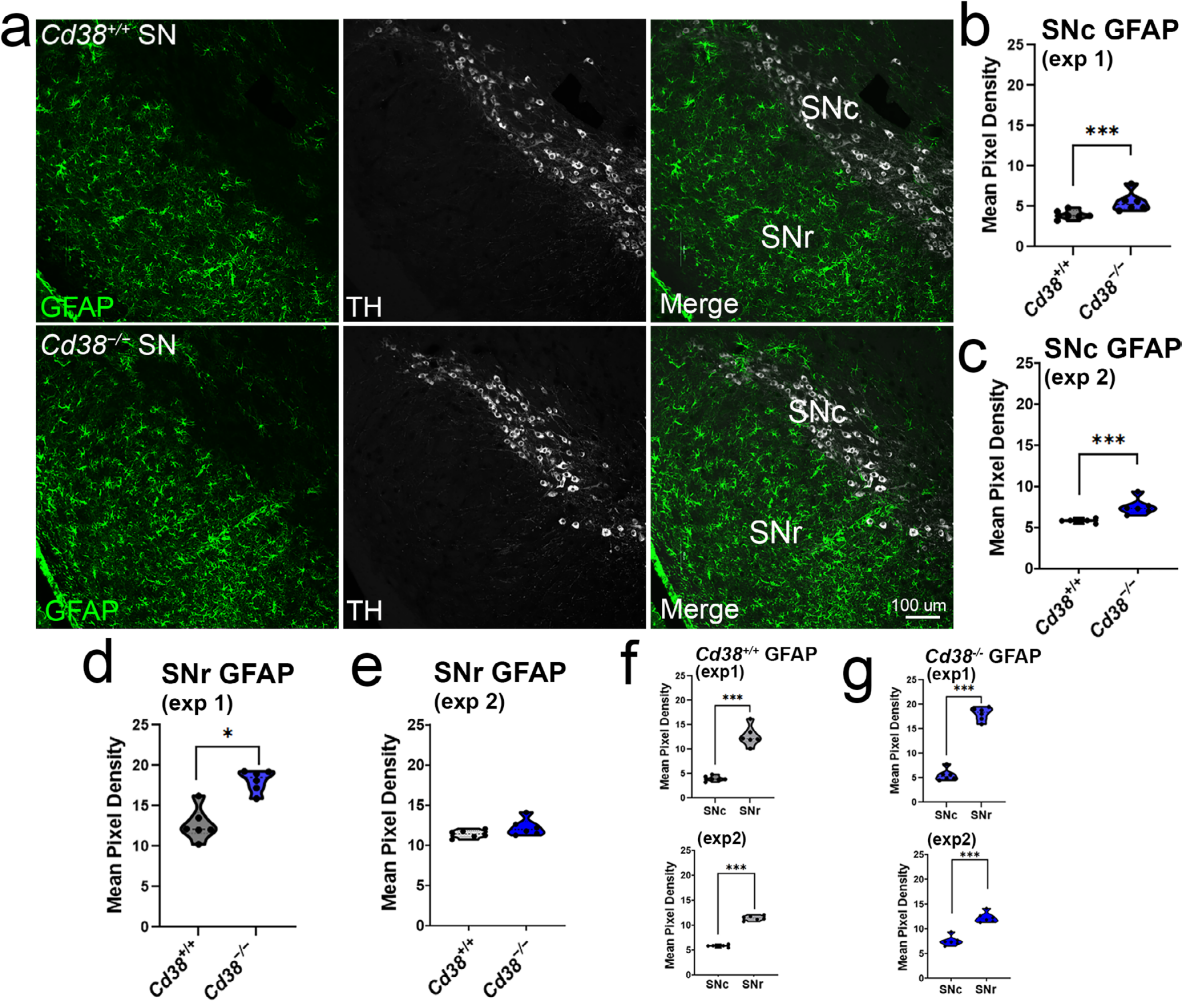
*Cd38* deficiency increases glial reactivity in the substantia nigra. (a) Representative images of immunofluorescence for GFAP and TH in *Cd38* ^*+/+*^ and *Cd38* ^*−/−*^ sections of substantia nigra pars compacta (SNc) and pars reticulate (SNr). (b) Nested *t*‐test revealed significantly higher mean pixel density of GFAP in the SNc in *Cd38* ^*−/−*^ compared to *Cd38* ^*+/+*^ in two experiments (b: *p* = 0.0008 and c: *p* = 0.0152) with variable expression in the SNr in two experiments (d: *p* = 0.0156 and e: *p* = 0.38). *n* = 3 *Cd38* ^*+/+*^ and *n* = 3 *Cd38* ^*−/−*^;3 nigral sections with 6 images per animal in both experiments. Nested *t*‐tests revealed significantly higher GFAP expression in the SNr compared to the SNc in two experiments in both *Cd38* ^*+/+*^ (f: *p* = 0.0004, *p* = 0.0002), and *Cd38* ^*−/−*^ (g: *p* = 0.0003, *p* = 0.0007) mice.

Based on astrocyte enrichment of CD38 and evidence for a regulatory role in inflammatory processes (Amici et al. 2018; Lischke et al. 2013; Musso et al. 2001), we sought to evaluate the effects of CD38 deficiency on the phenotypic states of astrocytes. We identified transcriptional changes in genes related to astrocyte inflammation and reactivity, notably increased expression of *C4b*, *Gfap*, and *Aqp4*, further suggesting *Cd38* deletion may impact the phenotypic state of astrocytes. To further explore the functional consequences of these transcriptional changes in vivo, we performed immunofluorescence for GFAP, a marker of astrocytic inflammation and reactivity, in the substantia nigra of 9–12‐month‐old *Cd38* ^*+/+*^ and *Cd38* ^*−/−*^ mice (Figure 6a). We analyzed astrocytes in their respective regions of the substantia nigra (pars compacta: SNc; pars reticulata: SNr). Nested *t*‐test analyses of immunofluorescence revealed increased mean pixel density of GFAP in the SNc of *Cd38* ^*−/−*^ mice in two separate experiments (Figure 6b,c), with more variable results in GFAP immunoreactivity in the SNr (Figure 6d,e). This inconsistency in the SNr between experiments could be due to rostral‐to‐caudal sampling differences, as our results indicated a robust enrichment in GFAP immunoreactivity in the SNr versus SNc of the midbrain of both *Cd38* ^*+/+*^ (Figure 6f) and *Cd38* ^*−/−*^ animals (Figure 6g). To determine whether the increases in inflammatory markers observed were due to changes in astrocyte number, we measured SOX9 counts in the SN and found no significant differences between the genotypes (Figure S3). These data indicate that deletion of CD38 causes changes in astrocyte reactivity state in the proximity of dopaminergic neuron cell bodies in the SN, with no changes in astrocyte numbers.

### 3.5 Differentially‐Expressed Transcripts in *Cd38*‐Deficient Mice Overlap With Genes Involved in Parkinson's Disease, Alzheimer's Disease, and Senescent State of Astrocytes

**FIGURE 7:**
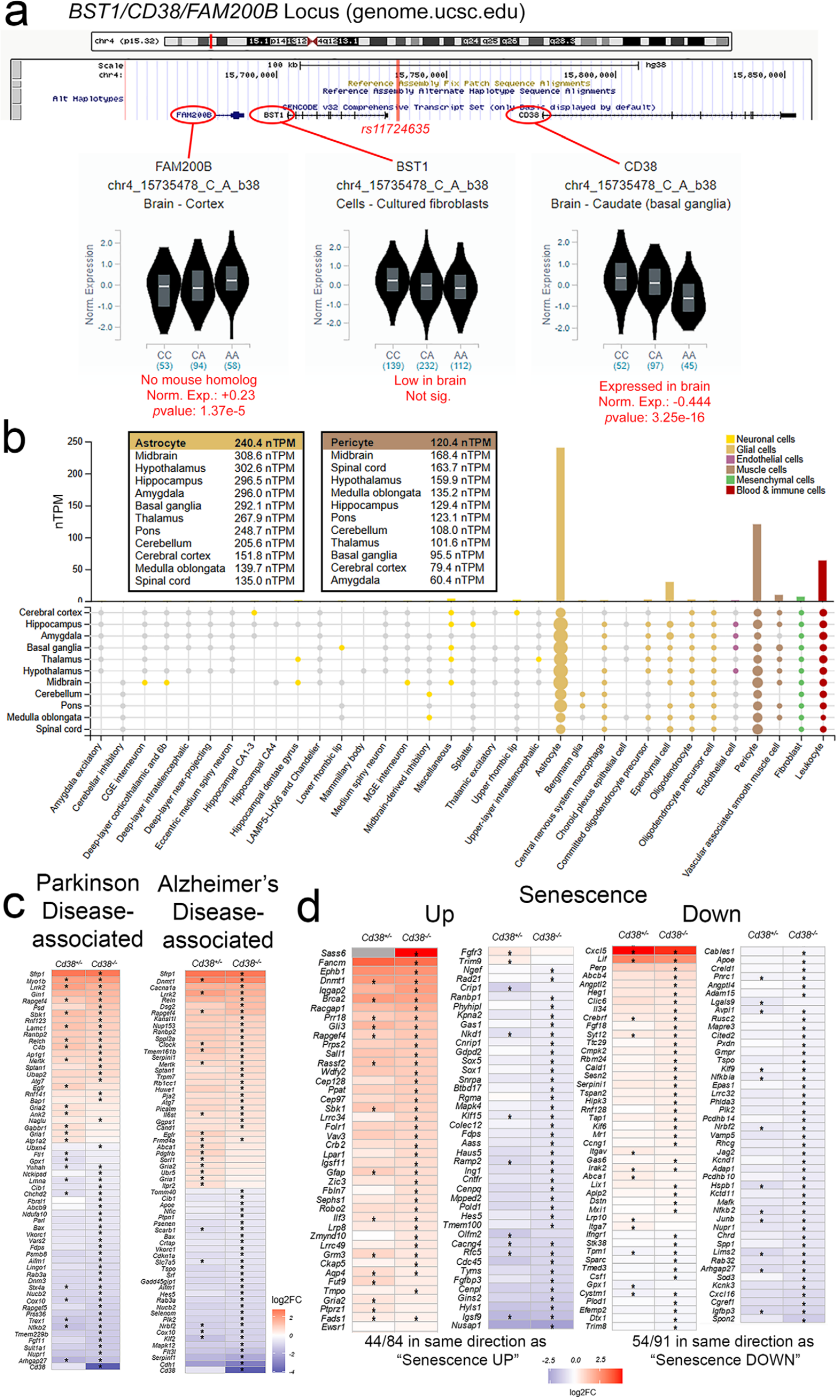
Relevance of *CD38* deficiency for pathobiological processes in Parkinson's disease and Alzheimer's disease. (a) *rs11724635* (top, red line) is located in the FAM200B/BST1/CD38 locus; individuals homozygous for the A/A PD risk allele exhibit increased expression of *FAM200B* (cortex as an example; left), no change in *BST1* (middle), and decreased expression of *CD38* (basal ganglia; right) (Kent et al. 2002). (b) Single‐cell transcriptional data from the Human Protein Atlas (Li et al. 2019), (c‐d) DEGs from Cd38+/‐ and Cd38‐/‐ vs. Cd38+/+ astrocytes overlap with genes associated with PD, AD (c) and senescence (d).

Considering recent studies suggesting that CD38 biology may be potentially useful to understand PD risk, we sought to determine whether alterations in *Cd38* expression are associated with the PD risk allele *rs11724635* (C effect allele: OR 0.89; A effect allele: OR 1.1) identified by others (Chang et al. 2017; Nalls et al. 2019). We explored associations between *rs11724635* and the expression of nearby genes *FAM200B*, *BST1* (upstream), and *CD38* (downstream) (Figure 7a) using the GTEx Portal (v. 8) (Chen et al. 2014; Li et al. 2019; Sharma et al. 2012). Notably, the A allele of *rs11724635* is associated with decreased *CD38* expression in the brain (Figure 7a, right, Figure S1), with no significant changes in *CD38* gene expression in non‐nervous system tissues (Figure S1). There were no associations observed between *BST1* expression and genotype in brain tissues, but there was increased *FAM200B* expression in brain tissues of A/A carriers (no mouse homologue). Evaluation of *CD38* expression by cell type in publicly available datasets (Allen Brain Atlas 2006; Karlsson et al. 2021; Kelley et al. 2018; Uhlén et al. 2015) revealed enrichment of *CD38* in astrocytes throughout the brain (mean expression: 240.4 nTPM), with the highest abundance in astrocytes of the human midbrain (308.6 nTPM) (Figure 7b) These data indicate that, similar to mice, humans show an enrichment of *CD38* expression in astrocytes with respect to other cell types.

Mitochondrial dysfunction is highly associated with PD; therefore, we sought to determine if our datasets indicate mitochondrial perturbations. Utilizing the MitoCarta 3.0 database (Rath et al. 2021), we evaluated our DEGs against those established to be directly involved with mitochondrial function. *Cd38* ^*+/−*^ and *Cd38* ^*−/−*^ comparisons indicated that 18 and 114 DEGs, respectively, are implicated in mitochondrial function (Figure S1). Shared amongst identified genes are those that mediate fatty acid synthesis (*Acacb*), complex IV activity (*Cox10*), protein synthesis (*Mrpl41*), complex I activity (*Ndufb7*), and mitochondrial DNA regulation (*Top1mt*). Dysregulation of complex I and IV genes is directly tied to mitochondrial ATP production, implicating mitochondrial bioenergetics may be perturbed. Given that CD38 function is implicated in PD (Zhou et al. 2023), astrocyte function (Hattori et al. 2017; Hayakawa et al. 2016), cADPR‐induced inflammation (Wei et al. 2014), and NAD‐related bioenergetics (Lautrup et al. 2019), we sought to cross‐reference our DEG findings with available datasets. First, we examined our pairwise comparisons relative to PD TWAS, GWAS, and PWAS datasets (Kia et al. 2021; Yao et al. 2021; Zhou et al. 2023). Numerous DEGs were matched to genes implicated in PD risk (Figure 7c, Table S2). Interestingly, both *Cd38* ^*+/−*^ and *Cd38* ^*−/−*^ astrocytes show increased expression of *Lrrk2*, a gene responsible for the majority of autosomal dominant PD cases in humans and another risk factor gene (Satake et al. 2009; Usmani et al. 2021). *Sod3* downregulation has also been implicated in human PD risk (Ilyechova et al. 2018) and was found to be downregulated in *Cd38* ^*+/−*^ and *Cd38* ^*−/−*^ comparisons.

Then, we compared DEGs with genes implicated by Alzheimer's disease (AD) GWAS studies (Beecham et al. 2014; Kamboh et al. 2012) (Figure 7c). Of note, *Apoe* was identified as downregulated in both comparisons. This finding is highly relevant to AD as changes in *APOE* expression are robust predictors of disease (Fernández‐Calle et al. 2022; Raulin et al. 2022). Furthermore, *Apoe* is implicated in astrocytic regulation of inflammatory responses (Lanfranco et al. 2021), blood–brain barrier integrity (Jackson et al. 2022), and neuronal excitability (Konings et al. 2021). Recently, astrocyte senescence has also been implicated in AD risk (Lau et al. 2023; Ungerleider et al. 2021). In comparison to genes identified as upregulated and downregulated in senescent astrocytes (Limbad et al. 2020), we found that 56% of DEGs shared the same directionality as previous observations (Figure 7d). Altogether, these results suggest that the transcriptional state induced by CD38 deficiency could inform mechanisms of astrocyte dysfunction in disease and senescence‐associated states.

## 4 Discussion

### 4.1 CD38 And the Biology of Astrocytes

The roles of CD38 in the CNS have been a growing topic of interest due to the proposed therapeutic potential of increasing NAD+ levels in aged persons. In physiologically healthy contexts, CD38 facilitates glial development (Hattori et al. 2017) and regulates NAD+/NAM balance (Aksoy et al. 2006). During aging, this balance is disrupted in part due to increased expression and activity of CD38 across tissues (Camacho‐Pereira et al. 2016; Guerreiro et al. 2020). Here, we demonstrate that, in the midbrain, *Cd38* is most highly expressed in astrocytes at higher abundance per cell than in another region of the basal ganglia, the dorsolateral striatum. Single‐cell studies (Figure 1a) suggest that other regions may express *Cd38* just as highly (globus pallidus), but the functional consequences of these differences are not known. Interestingly, while *Cd38* expression was lower per astrocyte in the striatum, NAD+/NAM ratios were similarly increased in both the midbrain and striatum of *Cd38* ^*−/−*^ mice, similar to what is seen in whole brain (Roboon et al. 2019) and peripheral tissues (Bu et al. 2018), suggesting that CD38 expression there is required for maintaining NAD+/NAM balance.

While these studies did not involve the deletion of *Cd38* selectively in astrocytes, based on its expression profile, these data suggest that NAD+ conversion to NAM is dependent on astrocytic expression of CD38 and that astrocytes, thus, are a strong contributor to NAD+/NAM metabolism in the brain. Particularly important to consider is the lack of *Cd38* mRNA expression in neurons, meaning that CD38‐dependent NAD+ to NAM conversion, while happening intracellularly in astrocytes, may not be occurring in neurons normally. However, CD38 is trafficked to the cell surface and can convert NAD+ to NAM in the extracellular space, providing NAM for local neurons. As such, NAM sources for neurons would be compromised in CD38 null mice, forcing neuronal dependence on intracellular sources (NAMPT, etc.) and possibly increasing neuronal vulnerability to oxidative stress, mitochondrial dysfunction, and other processes associated with neurodegeneration.

### 4.2 Mechanisms of Metabolic and Transcriptional Dysregulation

Our functional data in combination with our RNA sequencing data displaying altered transcriptomics in genes related to metabolism indicate CD38 deficiency impairs pyruvate oxidation, possibly due to changes in NAD+ metabolism, suggesting early metabolic dysregulation. Further, increased citrate synthase activity in 12‐month *Cd38* ^*−/−*^ indicates a potential increase of mitochondrial content in the absence of *Cd38* in an age‐dependent manner. Along those lines, we observed an increase in expression of *Mfn1*, the gene encoding Mitofusin 1, a protein involved in mitochondrial biogenesis. Additionally, normalization of COX to CS content indicates reduced complex IV activity in the 12‐month *Cd38*‐deficient mice. Yet, due to the nature of these experiments containing numerous cell types, we were unable to discern which cell types are driving the Complex IV deficit. Future studies evaluating CD38‐dependent mitochondrial changes within astrocytes are warranted.

Previous studies have demonstrated that CD38 deficiency can increase mitochondrial function (Camacho‐Pereira et al. 2016; Meyer et al. 2022). We propose that astrocyte metabolic state is influenced by disruption in the NAD+/NAM balance and reinforced by the transcriptional changes we have observed here. For example, the increase in citrate synthase activity (Figure 5) could be driven by the increased availability of NAD+ (Camacho‐Pereira et al. 2016; Lamb et al. 2020), independent of changes in transcriptional state. However, other NAD + ‐dependent processes could contribute to sustained changes in transcription, such as the activation of NAD + ‐dependent enzymes, including members of the PARP and sirtuin families. Considering the links between PARP activation/sirtuin inhibition and cellular senescence (Bai et al. 2011; Ohanna et al. 2011; Ponce et al. 2014) one may speculate that the overactivation of PARPs and/or sirtuins could contribute to shifts in the transcriptional state that mimic age‐related cellular senescence in the absence of CD38 (Chini et al. 2020).

### 4.3 Glial Reactivity in Substantia Nigra

We have shown that *Cd38* deletion enhances astrocytic activation and can influence the inflammatory state of astrocytes (Figures 3, 6). This enhanced inflammation has the potential to impact surrounding neurons. Interestingly, results were most consistent in the SNc with an increase in GFAP immunoreactivity. Dopaminergic neurons, which are selectively vulnerable in Parkinson's disease, are found in the SNc but extend their processes into the SNr, a region occupied by GABAergic neurons that provide strong inhibitory control via GABA receptors expressed on the dendrites of dopaminergic neurons. In the case of *Cd38* deletion, enhanced inflammation in the SNc and/or the SNr could influence dopaminergic neuron survival via astrocyte dysfunction surrounding neurons in the SNc, through disruption of the inhibitory cascade provided by GABAergic neurons in the SNr, or by activation of local microglia. Further data regarding the effects of *Cd38* deletion on dopaminergic neuronal survival are needed to better elucidate the role CD38 may play in neurodegenerative disorders like Parkinson's disease.

It is important to recognize that GFAP, a characteristic marker for activated astrocytes, was heterogeneously distributed in the midbrain with a high concentration of GFAP‐positive cell bodies in the SNr. In contrast, immunoreactivity for GFAP was lower in the SNc indicating subpopulations of astrocytes that differ substantially by subregion. Future studies should explore the properties of different types of astrocytes in the midbrain to fully elucidate the impact of CD38 deficiency on dopaminergic neuron function and viability.

### 4.4 CD38 and Neurodegeneration

There are conflicting results in the literature regarding the impact of CD38 deficiency on neuronal vulnerability. *Cd38*‐deficient mice are more sensitive to traumatic brain injury (Levy et al. 2009) and exhibit reductions in spine density and neuronal complexity, presumably during development (Hattori et al. 2023). In models of the autoimmune disease lupus, *Cd38* deletion accelerates disease progression, suggesting that CD38 deficiency can exacerbate inflammation (Viegas et al. 2011). However, *Cd38*‐deficient mice show decreased amyloid plaque load, microglial activation, and inflammation when crossed with hemizygous APPswePS1ΔE9 transgenic mice (Blacher et al. 2015), potentially due to decreased secretase activity in neurons. However, it is important to note that the contribution of cell‐autonomous effects in astrocytes to these findings is not known. In the context of amyloid processing, we found that *Apoe* expression is reduced in astrocytes of *Cd38* ^*−/−*^ mice; it is possible that the APOE deficiency in astrocytes could contribute to the reduced plaque load in these mice, due to APOE's roles in lipid metabolism and immune regulation (Kim et al. 2009; Ulrich et al. 2018).

While the pathology of PD has been well documented, there still exists a large gap in our current understanding of genetic and environmental risks that precipitate dopaminergic cell loss (Skrahina et al. 2021; Vollstedt et al. 2023) and the involvement of astrocytes in this process (Joe et al. 2018; Kam et al. 2020; Rizor et al. 2019). The SNP variant *rs11724635* has been implicated as a risk gene in multiple GWAS studies and meta‐analyses (Chen et al. 2014; Li et al. 2019; Sharma et al. 2012). In humans, the risk allele of *rs11724635* is associated with decreased *CD38* expression, although there is also a small reduction in expression of the human gene *FAM200B* (Figure 6). *FAM200B* has no mouse homologue, nor has its function been well characterized. Related to the possible impact of this risk variant on astrocyte function, it is important to note that the *Cd38* ^*+/−*^ mouse may be the best model to explore PD‐relevant phenotypes. While there were no alterations in tissue homogenate NAD/NAM ratios in *Cd38* ^*+/−*^mice, there were transcriptional and metabolic changes (Figures 3, 4 and 7c,d), similar to those observed in *Cd38* ^*−/−*^ mice. However, the most robust differences in the *Cd38* ^*+/−*^mice were transcriptional, with reductions in subsets of mitochondrial genes and increases in genes involved in glycogen metabolism (*Pygb*) and PD (e.g., *Lrrk2*). Increased expression of *Lrrk2* is of particular note, especially in light of recent reports indicating that AD patient‐derived iPSC astrocytes harboring the G2019S *LRRK2* mutation, associated with increased kinase activity, display a pro‐inflammatory phenotype as well as a decreased ability to promote angiogenesis (de Rus Jacquet et al. 2023). Considering these data and the low odds ratio for this SNP (~1.1), future studies can explore synergistic effects between CD38 deficiency and phenomena observed in PD such as synucleinopathy, inflammation, and/or oxidative stress.

Beyond this potential link to PD, it is important to consider age‐related processes, which could be impacted by CD38 deficiency. Comparisons of our dataset to aged‐striatal astrocytes indicate disruption in age‐related genes. This particular feature is of great interest as discordant expression of age‐related genes may suggest an advancement of age‐related processes (Frenk and Houseley 2018). We also found that ROS‐related astrocyte DEGs had reduced expression, which may be reflective of reduced inflammatory states in *Cd38*‐deficient models similar to reports by others (Roboon et al. 2019). DEGs common to both *Cd38* ^*+/−*^and *Cd38* ^*−/−*^ indicate that reductions in CD38 influence functions relating to metabolic oxygen utilization (*Cyp1b1*), antioxidant defense (*Nostrin, Sod3*), and inflammatory signaling (*Junb, Rora, C4b*). Across the aging process, ROS inflammatory drive is increased and contributes to neurodegenerative disease pathology (Zhang et al. 2023). We identified that ROS‐related genes are differentially expressed in *Cd38*‐deficient astrocytes. Based upon the established relationship of inflammation to PD development, one may suspect that reduced astrocytic inflammatory profiles by CD38 loss may be beneficial; however, this concept is not corroborated by human data (Ge et al. 2023; Yao et al. 2021; Zhou et al. 2023). While reductions in CD38 may appear to induce anti‐inflammatory states in astrocytes, they may instead be indicative of a loss of immunocompetence. Altogether, the benefits and risks associated with changes in CD38 presence and activity may be context‐dependent and require further investigation.

### 4.5 Limitations and Future Directions

Our findings suggest astrocyte dysfunction resulting from *Cd38* deficiency; however, there are limitations to this study that warrant recognition. The most immediate of which is the evaluation of dopaminergic neuron vulnerability in *Cd38*‐deficient mice, with relevance for PD. Future work will aim to understand how decreased CD38 expression in translationally relevant *Cd38* ^*+/−*^models affect DA neuronal gene expression, health, and function. It is also important for future work to explore how *Cd38* deficiency impacts transcription and metabolism in adult neurons.

Considering the high enrichment of CD38 expression in astrocytes, we are concluding that the results observed in astrocyte genes and metabolic changes in *Cd38* ^*−/−*^ homogenates are driven by cell‐autonomous effects in astrocytes. However, it is important to note that observed differences may be influenced by other cells, including those in the periphery that express CD38, and CNS cells surrounding astrocytes which typically express CD38, such as some populations of microglia and perivascular macrophages. Future experiments should involve the deletion of *Cd38* selectively in astrocytes of adult mice to differentiate developmental from dynamic CD38‐driven processes.

Age‐related changes are the leading risk for the development of neurodegenerative disease in humans. The conclusions drawn from our work rely upon “middle‐aged” animals (8–12 months), without inclusion of models of advanced age (18–24 months). Additional study of the interaction between aging and CD38 deficiency is warranted to help elucidate how age‐related changes contribute to astrocytic states. It is also important for future experiments to consider environmental contexts that may be translationally relevant to what is experienced by industrialized populations determined to be most at risk of PD (De Miranda et al. 2022). Paradigms that investigate interactions in genetically relevant models (*Cd38* ^*+/−*^) with environmental challenges such as high‐fat diet, chemical contaminants, or chronic inflammation may help establish better understanding of the intersectionality between gene risks and environmental events that instigate disease. Inflammation as a peripheral risk factor should be considered, in light of the expression of CD38 by cells of the peripheral immune system and evidence for a role of CD38 in pathogen clearance (Ghosh et al. 2023; Glaria and Valledor 2020; Lischke et al. 2013; Piedra‐Quintero et al. 2020).

Lastly, while not addressed directly here, it would be important to test the impact of clinically used CD38 inhibitors on brain metabolism and astrocyte function. CD38 monoclonal antibody (Isatuximab; (Bisht et al. 2023)) and inhibitors (Chini et al. 2018) are currently being used for treating patients with multiple myeloma and other cancers (Gao et al. 2021) and have been proposed for the treatment of neurodegeneration, based on the prediction that increasing NAD+ levels could be neuroprotective (Chini et al. 2018; Guerreiro et al. 2020). However, considering the localization of CD38 to astrocytes and our results here indicating cellular dysregulation even in the face of NAD+ abundance, other modes of NAD+ production should be considered. CD38 inhibitors could shift the metabolic state of astrocytes and cause depletion of neuronal NAD+ stores by reducing extracellular sources of NAM. Additional studies are required to test these predictions.

## 5 Conclusions

This work seeks to resolve how the decreased expression of the metabolic enzyme *Cd38* influences astrocytes. We demonstrate that deficiencies in CD38 affect astrocyte gene expression, metabolism, and molecular pathways associated with the hallmarks of neurodegeneration. These findings further our understanding of how CD38 facilitates astrocyte function in the aging brain, while simultaneously providing evidence for how its dysregulation may be tied to neurodegenerative features.

## Author Contributions

S.B.: experimental design, data collection and analysis, figure generation, manuscript editing; A.M.C.: experimental design, tissue collection, data collection and analysis, figure generation manuscript content creation and editing; R.D.H.: experimental design, data collection and analysis, figure generation, manuscript content creation and editing; X.W.: data analysis and figure design/creation; L.J.M.: experimental design, data collection, figure generation; D.D.: data collection and analysis; M.S.: experimental design, data collection and analysis; J.L.B.: figure creation and editing, K.N.: data collection; F.Z.: experimental design, data collection; F.E.L.: conceptual and experimental design; J.S.S.: experimental design, data collection, data analysis, figure generation; S.G.W.: data collection and analysis; A.M.P.: data analyses, manuscript content creation and editing; R.M.C. and M.L.O.: securing funding, experimental design, staff management, data analyses, figure generation, manuscript content creation and editing.

## Funding

This work was supported by NIH/NINDS R01NS124037 (RMC, MLO) and NIH/NINDS T32NS09.5775 Additionally, the High‐Field NMR facility at the University of Alabama at Birmingham was established through the NIH (1S10RR026478) and is supported by the UAB Comprehensive Cancer Center (NCI grant P30 CA013148).

## Ethics Statement

All mouse experimental protocols were followed according to NIH guidelines and approval from the Animal Care and Use Committee of Virginia Tech and the University of Alabama at Birmingham.

## Conflicts of Interest

The authors declare no conflicts of interest.

## Supporting information


**Figure S1:** Multi‐tissue eQTL plot from the GTEx Portal (v.8) for single nucleotide polymorphism associated with Parkinson's disease, with a shift to the left for carriers of the A allele (see Figure 6).

**Figure S2:** Differentially expressed genes associated with mitochondrial function in isolated astrocytes from *Cd38* ^*+/−*^ or *Cd38* ^*−/−*^ mice compared to *Cd38* ^*+/+*^ mice.

**Figure S3:** glia70112‐sup‐0003‐FigureS3.pdf. *Cd38* deficiency does not impact SOX9 nuclear expression.
(a) Representative images of immunofluorescence for SOX9 and TH in *Cd38* ^*+/+*^ and *Cd38* ^*−/−*^ sections of substantia nigra pars compacta (SNc) and pars reticulate (SNr), (b) No significant differences were found in the counts of SOX9‐positive cells between the two genotypes in the SNc region (*p* = 0.7798). An unpaired *t*‐test was performed for the measure between genotypes. *n* = 4, *Cd38* ^*+/+*^ *and n* = 5, *Cd38* ^*−/−*^ with two SN sections per animal.

**Table S1:** RNASeq TPM data all genotypes.

**Table S2:** Cd38+/+ vs. Cd38−/−.

**Table S3:** GO pathway analysis derived by pairwise comparisons between Cd38+/− to Cd38+/+ and Cd38−/− to Cd38+/+.

## Data Availability

All RNA‐Seq fastq reads can be found at GEO Series accession number GSE272765. The following secure token has been created to allow review of record GSE272765 while it remains in private status: **wlivemmeflennan**. Additional data that support the findings of this study are available on Mendeley Data with a reserved DOI: https://doi.org/10.17632/dpxwtw4xvz.1. This will be published upon acceptance for publication.
