# Supplementary figures and images for: Novel Roles for the Ectoenzyme CD38 in the Maintenance of Transcriptional and Metabolic Homeostasis in Astrocytes
Source: Glia. 2025 Dec 16;74(2):e70112. doi: 10.1002/glia.70112 (PMC12706826; doi:10.1002/glia.70112)

## Multi-tissue eQTL plot (rs11724635)

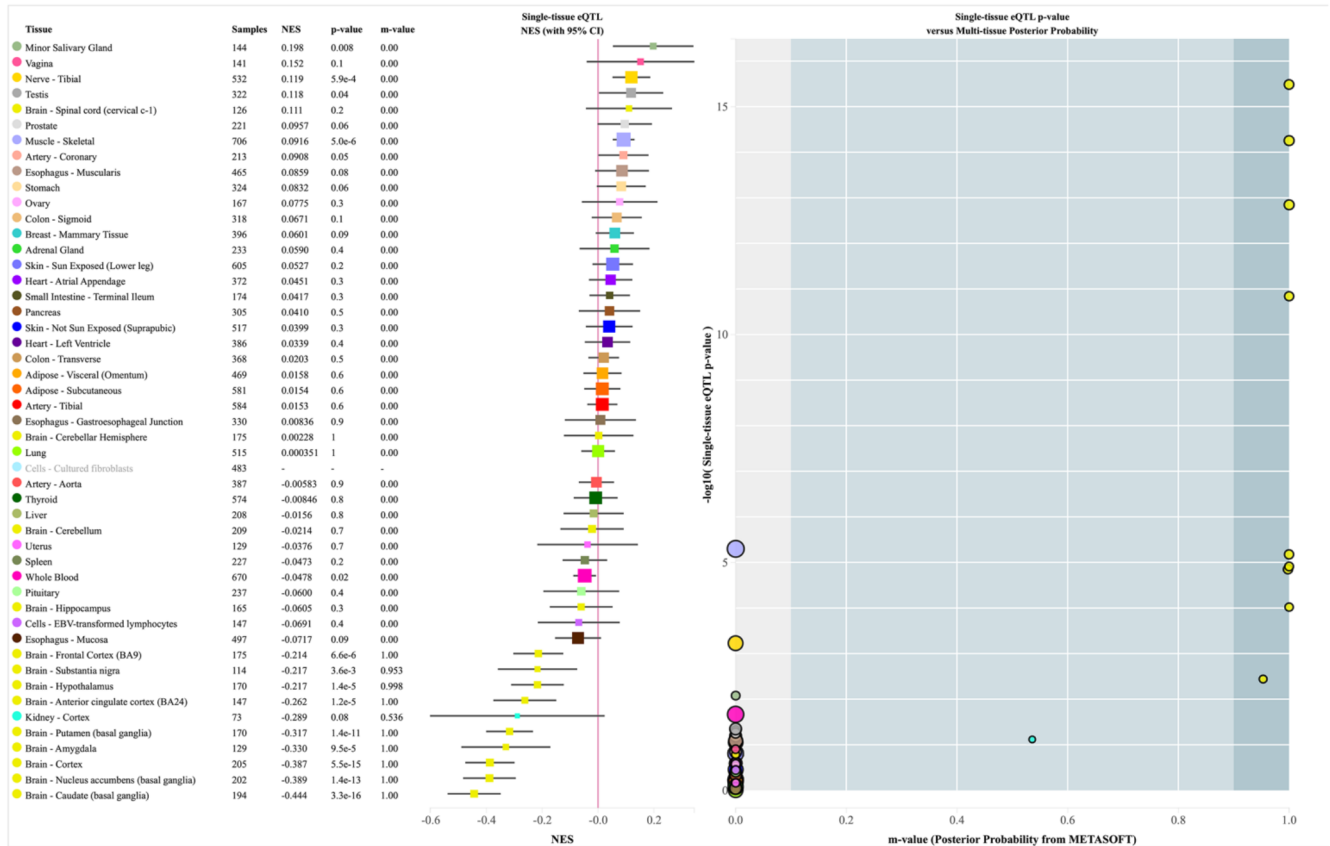

chr4\_15735478\_C\_A\_b38\_ENSG00000004468.12.multiTissue.svg

Supplement: Supplementary file 1 — Figure S1: Multi‐tissue eQTL plot from the GTEx Portal (v.8) for single nucleotide polymorphism associated with Parkinson's disease, with a shift to the left for carriers of the A allele (see Figure 6). [file GLIA-74-0-s001.pdf]

# Genes Associated with Mitochondrial Function

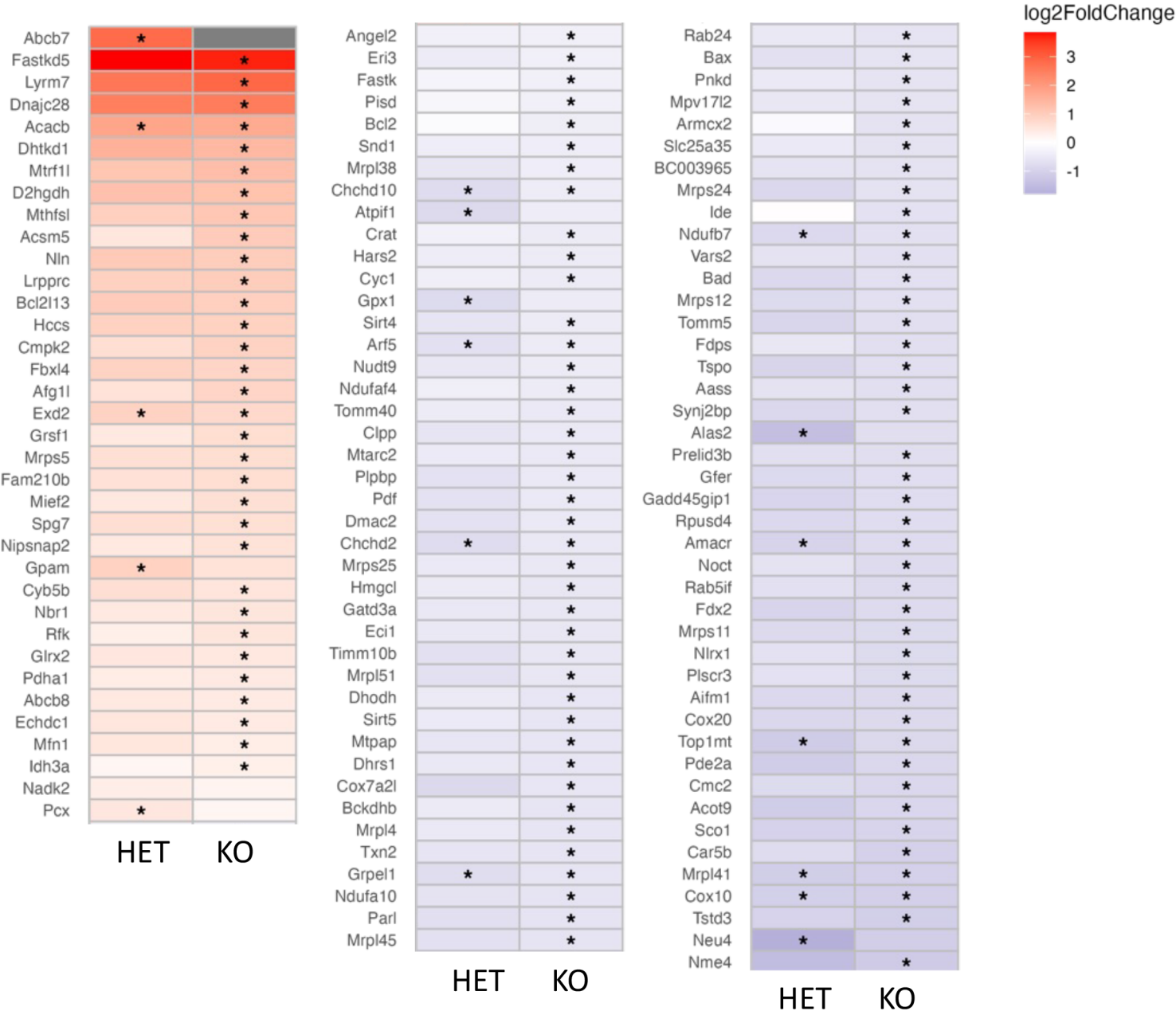

Supplement: Supplementary file 2 — Figure S2: Differentially expressed genes associated with mitochondrial function in isolated astrocytes from Cd38 +/− or Cd38 −/− mice compared to Cd38 +/+ mice. [file GLIA-74-0-s004.pdf]

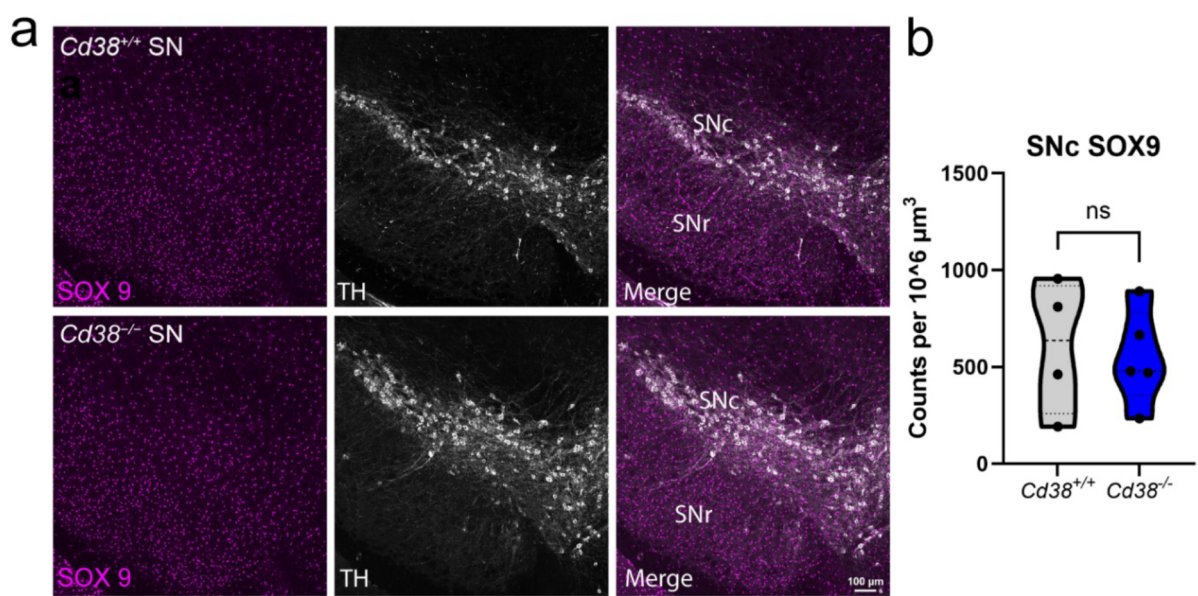

Supplement: Supplementary file 3 — Figure S3: glia70112‐sup‐0003‐FigureS3.pdf. Cd38 deficiency does not impact SOX9 nuclear expression. (a) Representative images of immunofluorescence for SOX9 and TH in Cd38 +/+ and Cd38 −/− sections of substantia nigra pars compacta (SNc) and pars reticulate (SNr), (b) No significant differences were found in the counts of SOX9‐positive cells between the two genotypes in the SNc region (p = 0.7798). An unpaired t‐test was performed for the measure between genotypes. n = 4, Cd38 +/+ and n = 5, Cd38 −/− with two SN sections per animal. [file GLIA-74-0-s006.pdf]
